# ESHRD: deconvolution of brain homogenate RNA expression data to identify cell-type-specific alterations in Alzheimer’s disease

**DOI:** 10.18632/aging.102840

**Published:** 2020-03-02

**Authors:** Ignazio S. Piras, Christiane Bleul, Joshua S. Talboom, Matthew D. De Both, Isabelle Schrauwen, Glenda Halliday, Amanda J. Myers, Geidy E. Serrano, Thomas G. Beach, Matthew J. Huentelman

**Affiliations:** 1 Neurogenomics Division, The Translational Genomics Research Institute, Phoenix, AZ 85004, USA; 2 Center for Statistical Genetics, Department of Neurology, Gertrude H. Sergievsky Center, Columbia University Medical Center, New York, NY 10032, USA; 3 The University of Sydney School of Medicine, Sydney, Camperdown NSW 2050, Australia; 4 University of Miami, Miami, FL 33124, USA; 5 Banner Sun Health Research Institute, Sun City, AZ 85351, USA

**Keywords:** laser capture microdissection, RNA sequencing, brain homogenates, endothelial cells, oligodendrocytes

## Abstract

Objective: We describe herein a bioinformatics approach that leverages gene expression data from brain homogenates to derive cell-type specific differential expression results.

Results: We found that differentially expressed (DE) cell-specific genes were mostly identified as neuronal, microglial, or endothelial in origin. However, a large proportion (75.7%) was not attributable to specific cells due to the heterogeneity in expression among brain cell types. Neuronal DE genes were consistently downregulated and associated with synaptic and neuronal processes as described previously in the field thereby validating this approach. We detected several DE genes related to angiogenesis (endothelial cells) and proteoglycans (oligodendrocytes).

Conclusions: We present a cost- and time-effective method exploiting brain homogenate DE data to obtain insights about cell-specific expression. Using this approach we identify novel findings in AD in endothelial cells and oligodendrocytes that were previously not reported.

Methods: We derived an enrichment score for each gene using a publicly available RNA profiling database generated from seven different cell types isolated from mouse cerebral cortex. We then classified the differential expression results from 3 publicly accessible Late-Onset Alzheimer’s disease (AD) studies including seven different brain regions.

## INTRODUCTION

Single cell gene expression changes are critical to fully understand neurological disease due to the significant cellular heterogeneity within the brain. However, the generation of such data requires very labor- and cost-intensive approaches (like laser capture microdissection, LCM, or single-cell RNA sequencing, scRNA-Seq) typically to the detriment of overall sample size and statistical power. For these reasons, most of the gene expression profiling studies on post-mortem brains from Alzheimer’s disease (AD) patients have been conducted on tissue homogenates. However, several LCM studies on AD have been undertaken on microglia [1, 2], astrocytes [3] and neurons [4], but not in endothelial cells, oligodendrocytes, or any other cell type in the brain. Unsurprisingly, these LCM studies are relatively limited in their sample sizes (with all of them including less than 48 total samples). A study conducted using scRNA-seq on brain cell nuclei in AD cases and controls was recently published [5]. The authors successfully sequenced individual nuclear RNA of 48 individuals (24 with AD pathology, and 24 without pathology) from 6 brain cell types (including oligodendrocytes), but excluding pericytes and endothelial cells due to the low cell counts in their resulting data.

The study of more extensive sized sample collections is of great importance in AD due to the neuropathological (e.g., Braak and Consortium to Establish a Registry for Alzheimer's Disease - CERAD scoring), genetic (e.g., APOE carriers vs. non-carriers), and demographic (e.g., age-at-onset, race, ethnicity, and sex) variability observed across patients with the disease. Therefore, while LCM and scRNA-Seq studies directly address the cellular heterogeneity in brain tissue, their typical small sample sizes result in a general inability to explore some of the other variables of interest in AD due to power concerns. Ultimately, the overall goal of gene expression profiling in AD is to understand the transcriptome changes in all major cell types of the brain in a well-powered approach that would facilitate the exploration of all the variables mentioned above.

Microglia are of interest due to their association with neuroinflammation in AD which has been shown to influence cognition [6]. Astrocytes are involved in brain homeostasis, and in AD they are linked to the production, degradation, and removal of Amyloid-β (Aβ). Astrocytes may also be included in the oxidative stress, and inflammatory changes noted in AD [7]. Neurons in the AD brain are characterized by synaptic loss, but also by defective neurogenesis, presumably caused by the abnormal accumulation of Aβ oligomers [8]. Endothelial cell dysfunction is also noted during the pathogenesis of AD. For instance, Amyloid plaque deposition and neurofibrillary tangle production may in part be due to the result of hypoxia due to the inadequate blood supply to the brain in AD [9]. The “vascular hypothesis” for AD argues that the cerebral hypoperfusion, resulting from vascular disease and aging, leads to the neuronal death and cognitive dysfunction in AD [10, 11]. Finally, oligodendrocytes may play a role in neurodegeneration due to the lower rate of re-myelination during aging, possibly due to age-related DNA damage in these cells which seem to be more vulnerable than others to oxidative stress [12].

In addition to previously published approaches [13, 14], we describe herein a bioinformatics approach that can leverage expression profiling data from brain homogenate (or “bulk”) tissue to derive cell type-specific differential expression and pathway analysis results. We applied our approach to different gene expression datasets derived from brain homogenate profiling from AD patients and Non-Demented controls (ND) from 7 different brain regions. We demonstrate the ability of this approach to highlight known neuronal-specific changes in the AD brain and utilize it to identify novel changes in endothelial cells and oligodendrocytes, two cell types not easily examined in the brain and for which only minimal gene expression knowledge exists in AD.

## RESULTS

### Proportion of cell-specific genes in the database

The proportions for “mixed” and cell-specific genes in the public database from mouse cerebral cortex we utilized to define the ESHRD (Enrichment Score Homogenate RNA Deconvolution) are reported in Supplementary Figure 1. Differentially Expressed Genes (DEGs) we labeled as “mixed” represent the most prevalent class (73.4%), followed by DEGs labeled as microglia (6.6%), neuron (5.9%) and endothelial (5.7%). Astrocyte and oligodendrocyte labeled DEGs have a frequency of 3.6% and 3.1%, respectively.

### Method validation

We used a dataset of Multiple System Atrophy (MSA) patients (*n* = 4) and controls (*n* = 5) to validate our ESHRD method. We conducted RNA expression profiling from both brain homogenates and oligodendrocytes obtained by LCM from the same donor brains and then calculated differential expression. We compared the concordance rate of the log2 Fold Change (FC) between the two analyses, filtering for different cutoffs (from False Discovery Rate - FDR < 0.05 to FDR = 1.00 with a 0.001 step-wise increase), and selecting the overlapping oligodendrocyte specific genes. We observed a 100% concordance rate for all of the FDR cut-offs ≤ 0.280, and > 80% for FDR < 0.511. The concordance rate decreases to 60.6% when we do not apply any FDR filtering. The ρ Spearman’s correlation coefficient was variable when we compared less than 20 genes, and ranged from 0.227 to 0.400 for larger sets of compared genes, becoming statistically significant at FDR < 0.579 (*n* = 37 genes) (Supplementary Figure 2).

Furthermore, we compare our results with the snRNA-seq study from Mathys et al. [5]. We downloaded the complete differential expression results (comparison: pathology vs no-pathology), using the log2 FC of the cell-level analysis including only genes with different log2 FC cutoffs (log2 FC = 0, 0.25, 0.50, and 1.00) without filtering for p-value aiming to include a larger number of genes. We considered only microglia and astrocytes for a direct comparison with our data. In the snRNA-seq study there were two classes of neuronal genes (excitatory and inhibitory) and two classes of oligodendrocytes which was not possible to characterize using our classification approach. Therefore, neurons and oligodendrocytes were excluded from this comparison. We compared these lists with the DEGs obtained from the 7 brain regions we classified as cell specific genes for microglia (*n* = 487) and astrocytes (*n* = 318), and we evaluated the log2 FC concordance. Excluding the brain regions and cells for which we obtained little overlap (less than 10 overlapping genes), we observed a concordance rate ranging from 58.9% (parahippocampal gyrus: PHG) to 87.5% (dorsolateral prefrontal cortex: DLPFC) for astrocytes, and from 57.5% (cerebellum: CBE) to 84.6% (superior temporal gyrus: STG) for microglia (Supplementary Table 1).

### Prevalence of DEGs among cell types and regions in Alzheimer’s disease brains

**Figure 1:**
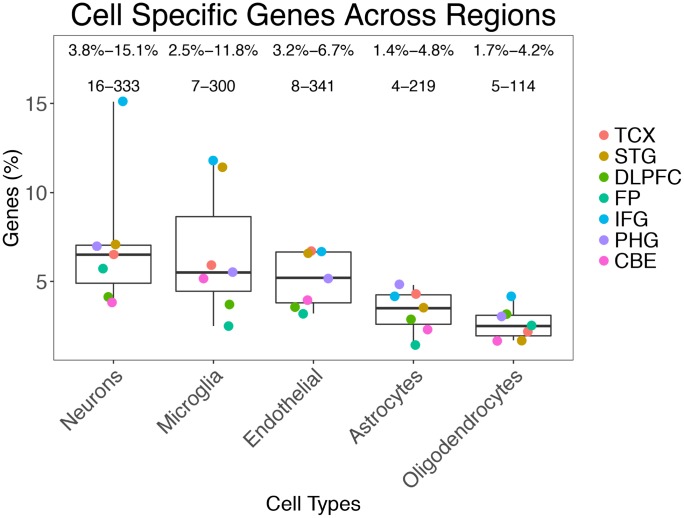
**Prevalence of gene classes expressed in different cells across the brain regions analyzed.**

The number of DEGs in the AD datasets ranged from 175 (in the inferior frontal gyrus: IFG) to 7,030 (temporal cortex: TCX) (Supplementary Figure 3). Using ESHRD, we classified all of the DEGs for a total of 15,806 genes, with 8,969 unique gene symbol (several genes were differentially expressed in multiple regions). We detected 32 different classes of genes based on their cell type specificity. Besides the “mixed” gene class, and the five cell-specific classes, we detected 26 classes where the expression was relevant in more than one cell type (Supplementary Table 2). When considering all of the DEGs across the brain regions represented in the selected AD studies, the “mixed” gene class were the most represented (*n* = 11,964; 75.7%), followed by neuron (*n* = 916; 5.8%), microglia (*n* = 885; 5.6%), endothelial (*n* = 830; 5.3%), astrocyte (*n* = 581; 3.7%) and oligodendrocyte classes (*n* = 375; 2.4%). The prevalence of genes expressed in single cell types show a mostly consistent pattern across the brain regions. Of note, neuronal genes are the most represented class of DEGs in 5 brain regions, but not in TCX and CBE, where the most represented DEGs classes are endothelial and microglia, respectively (Figure 1).

### Log2 FC distribution of gene classes

**Figure 2:**
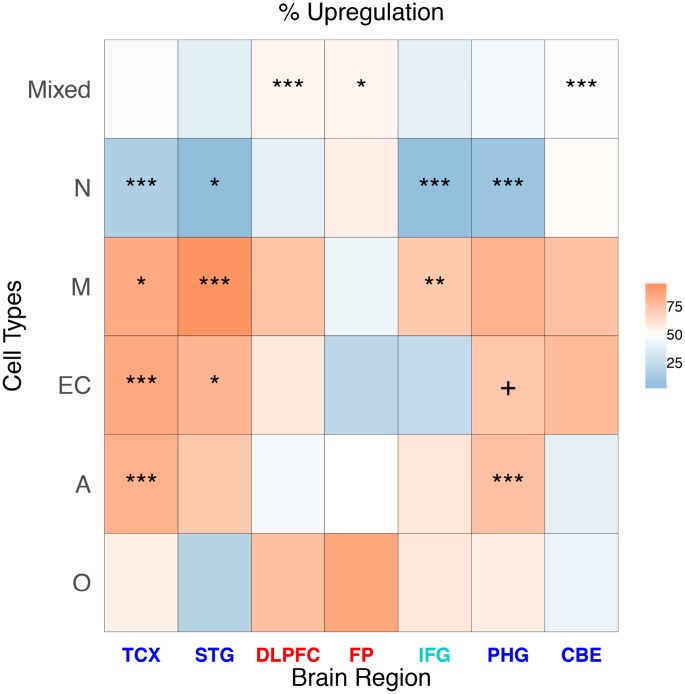
**Heatmap representing the proportion of up-regulated AD genes for cell type and brain region in cell-specific and “mixed” genes.** The stars represent a significant enrichment of a particular gene type among DEGs.

We report the log2 FC distribution of the genes represented in the single cell classes in each brain region (Table 1), representing the results as a heatmap (Figure 2)*.*

**Table 1:** Percentage of upregulation (% of genes with log2 FC > 0 and FDR < 0.05) for each brain region and cell type. In brackets, the number of DEGs classified is reported.

**Cell**	**TCX (5,514)**	**STG (594)**	**DLPFC (1,363)**	**FP (282)**	**IFG (119)**	**PHG (4,123)**	**CBE (4,211)**
**Mixed**	47.4	38.0	54.4	54.8	38.8	44.6	48.8
**N**	16.2	4.8	39.3	56.3	5.6	9.3	51.6
**M**	83.7	92.6	74.0	42.9	71.4	81.0	74.1
**EC**	84.5	79.5	59.2	22.2	25.0	72.2	76.8
**A**	80.8	71.4	46.2	50.0	60.0	74.5	39.2
**O**	56.1	20.0	75.0	85.7	60.0	57.6	41.4

Neuronal class DEGs are consistently downregulated in all of the brain regions except frontal pole (FP) (where 56.3% of Neuron DEGs are upregulated) and CBE (51.6% are upregulated). The result for FP was confirmed when we used less conservative FDR cutoffs to include a larger number of genes (Supplementary Figure 4). Microglia class DEGs are upregulated in all regions except FP (where only 42.9% of microglia DEGs are upregulated). Endothelial cell-specific DEGs are upregulated in all regions, except FP (22.2% are upregulated) and IFG (25.0% are upregulated). The astrocyte-specific DEG class is upregulated in TCX, STG, inferior frontal gyrus (IFG), and PHG, but demonstrates a downregulation in other regions, especially in CBE. Finally, we observed the oligodendrocyte DEG class as upregulated in all of the regions (especially in DLPFC and FP) except STG and CBE.

In some cases, the prevalence of cell type-specific genes among the DEGs was higher than expected by chance. We assessed this association by permutation analysis, and we report the results in the heatmap in Figure 2. The neuron gene class is significantly prevalent in more regions (TCX, STG, IFG, and PHG) followed by the microglia gene class (enriched in TCX, STG, and IFG).

### Cell-specific pathways analysis

**Figure 3:**
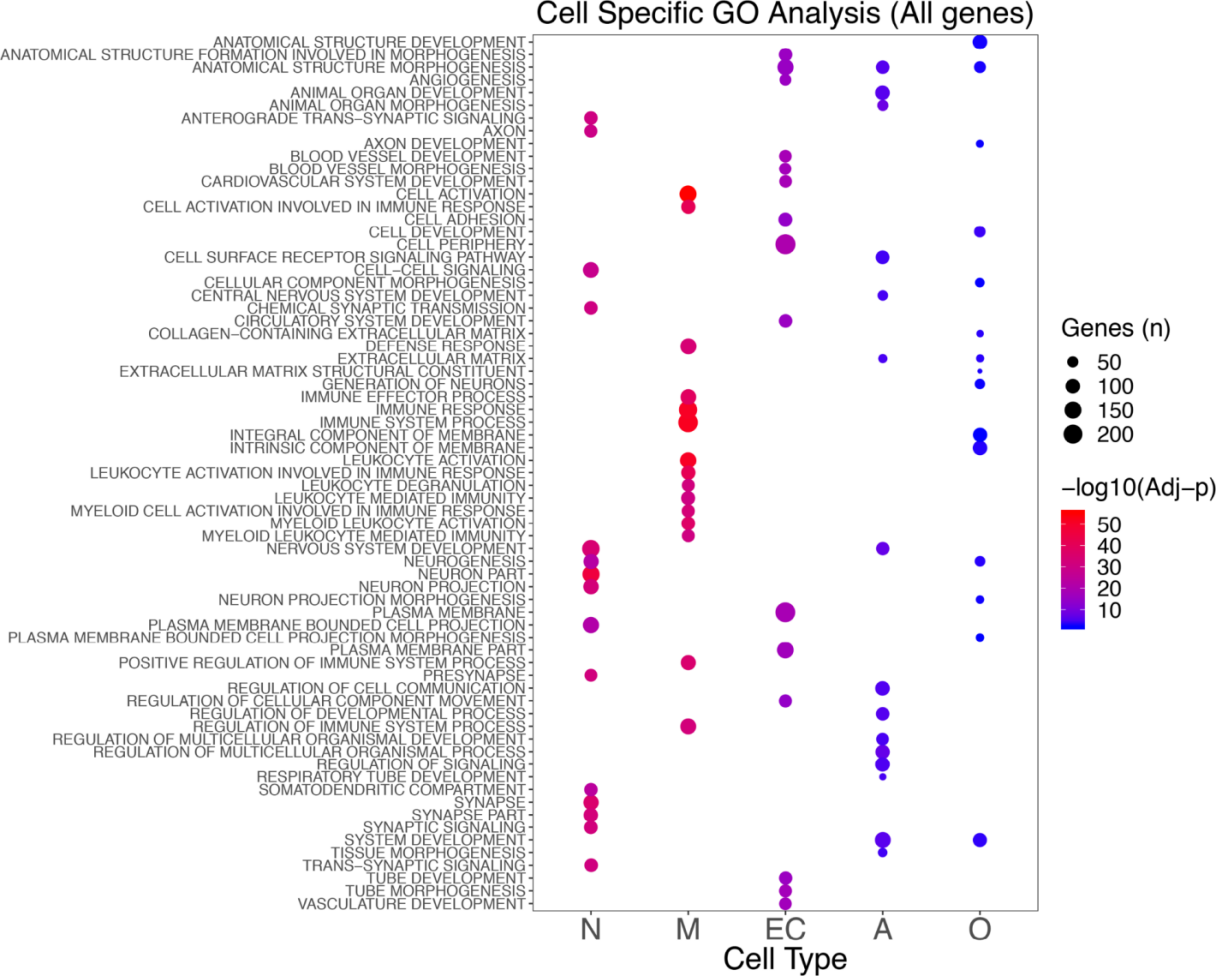
**Top 15 significant GO classes identified in the different cell types combining the results for the seven brain regions analyzed.** The color scale indicates the significance (blue to red as the significance increases), whereas the size shows the number of genes in that specific enriched class in AD.

Using Gene Ontology (GO), we identified significantly enriched processes for each cell type (Supplementary Table 3A–3C; Supplementary Figure 5). Genes showing the most specific functional classes were neurons (synaptic and neuronal), endothelial (vascular and angiogenesis), and microglia (immune system). Astrocyte genes were enriched in developmental processes and cell-cell signaling, whereas oligodendrocytes were enriched for developmental and neurogenesis processes (Figure 3).

Examining only the upregulated genes, we did not observe any enrichment for neuronal genes, but we confirmed almost all of the processes found in microglia, endothelial cells, and astrocytes in the total analysis. When using only the downregulated genes, we established the enrichment for neuron-specific genes, and we observed enrichment for postsynaptic membrane classes in oligodendrocytes (Supplementary Figure 5).

When we used the REACTOME database, we confirmed the pathways detected with the GO analysis (Supplementary Table 4A–4C). Furthermore, we identified the “GPCR ligand binding” pathway in neuronal cell-specific genes and platelet-related pathways in endothelial cells. In microglia, we confirmed the immune pathways, with strong enrichment for “Neutrophil degranulation” and several classes of Toll-Like receptor cascades. Finally, in oligodendrocyte specific upregulated genes we found enrichment for glycosaminoglycan pathways including : “Chondroitin sulfate/dermatan sulfate metabolism,” “Disease of glycosylation”, and “Syndecan interactions” (Supplementary Figure 6).

### Brain region cell-specific pathway and GSEA analysis

Using GO, we were able to detect significantly enriched processes in all the regions except DLPFC and FP. In all of the remaining regions we detected enrichment for microglia upregulated genes for immune processes, whereas the neuronal downregulated genes were enriched for synaptic processes in TCX, STG, and PHG. Endothelial genes were enriched for “Angiogenesis” and “Plasma membrane” classes in TCX and CBE, whereas in PHG the same genes were mostly enriched for “Cell signaling” and “Extracellular matrix” processes. Astrocyte were enriched for Developmental processes and Neurogenesis in TCX and PHG. Finally, oligodendrocytes were enriched for “Extracellular matrix” and “Postsynaptic membrane” in TCX (Supplementary Table 5; Supplementary Figure 7)**.**

To further explore the brain region-specific enrichment, we used Gene Set Enrichment Analysis (GSEA). The most relevant findings not detected in the previous analysis were for astrocyte genes, with the enrichment of “Innate immune system” in FP and PHG. We also observed several classes related to GPCR receptors in the IFG for neuronal genes (also detected with REACTOME analysis using the whole list of genes). Finally, we confirmed the enrichment for the process “Disease of glycosylation” in oligodendrocytes in TCX (Supplementary Table 6 and Supplementary Figure 8).

## DISCUSSION

### Overview

We propose herein a bioinformatics method termed ESHRD to derive cell type-specific differential expression and pathway analysis results by leveraging expression profiling data from brain homogenates. We applied ESHRD to different large RNA profiling datasets in AD that included many different brain regions. This approach allowed us to identify classes of genes expressed in specific cell types, and functionally related for processes not identifiable using the complete expression information obtained from the “bulk” tissue analysis.

### Homogenate, LCM and snRNA sequencing comparison using ESHRD

To examine the ability of the ESHRD approach to estimate cell type-specific gene expression results we compared the data obtained from brain homogenates and oligodendrocytes obtained by LCM from MSA cases and controls. We demonstrated that by using brain homogenates and ESHRD, we could detect 85.1% (n = 109) of oligodendrocyte genes found in the laser-captured oligodendrocytes. Furthermore, the log2 FC concordance between differential analyses was always larger than 60% regardless the FDR cutoff, with values ranging from 80% to 100% for FDR < 0.511, demonstrating that the DEG results we calculated in homogenates using ESHRD are in high concordance with what we observe after LCM. Additionally, the comparison of the DEGs classified as microglia or astrocytes from AD data from a recent snRNA-seq study [5] yielded a concordance rate from 58.8% (CBE, microglia) to 88.9% (STG, astrocytes).

### Neuronal, endothelial cells, and microglia are the most represented “cell-specific” gene classes in AD brain

The “mixed” genes, those not attributable to specific cells, were the most represented among DEGs (75.7%). This finding isn’t surprising as one may expect a high majority of genes to be shared in expression across cell types as they presumably encode proteins that perform core functions for all cells such as metabolism. The most “cell-specific” gene classes represented were neurons (5.8%), microglia (5.6%), and endothelial cells (5.3%). These same three types of cell-specific genes are the most represented across all of the brain regions we examined. The least designated class was oligodendrocytes (Total: 2.4%). The neuron-specific DEG class was the most prevalent class in all of the examined brain regions except STG and CBE. In those two regions, the most represented class of DEGs were microglia specific genes. Neuronal genes were significantly overrepresented among the DEGs in 4 regions (TCX, STG, IFG, and PHG).

The proportion of cells types, and especially the overrepresentation of the neuronal cell-specific class, is probably correlated to the differential cellular susceptibility to AD. The neuron/glia ratio estimates for the whole brain range from 1:10 [15–17] to 1:1 [18]. The prevalence of gene classes we detected may reflect the main processes present in AD: neurodegeneration [19] (neurons), neuroinflammation [20] (microglia) and brain vascular changes [21] (endothelial cells).

### Neuronal specific genes are downregulated and enriched for synaptic processes in AD

The neuronal DEGs are consistently significantly downregulated in all of the brain regions of AD patients, except FP (56.3% are upregulated) and CBE (51.6% are upregulated). The result found for FP might be biased by the small number of neuronal genes detected (*n* = 16), a consequence of the overall low number of DEGs observed in this brain region. However, the same result was confirmed and strengthened when we used less conservative cutoffs aiming to include a larger number of neuronal genes. Hyperexcitability of neurons was observed to be promoted by amyloid-β1–42 [22, 23]. Busche et al. observed an increased number of hyperactive neurons in the hippocampal CA1 subregion of young APP/PS1 transgenic mice suggesting that soluble Aβ oligomers may directly induce neuronal hyperactivity [24], contributing to malfunction of hippocampal circuitry and causing memory impairment [25, 26]. Finally, Ciccone et al. found a key role of Nav1.6 channels as determinant for hippocampal neuronal hyperexcitability [27]. Perhaps these mechanisms are at play in the FP and may be resulting in the neuron-specific gene upregulations.

Our results are in agreement with a recent snRNA-seq study from the prefrontal cortex [5], in which the two types of neurons investigated (inhibitory and excitatory) show dysregulation in AD (75% in inhibitory and 95% in excitatory neurons). This result demonstrates how the disease may potentially affect the neuronal cells across many brain regions, and this is especially true in the temporal lobe and the hippocampus – areas affected earlier in the pathological progression of the disease in comparison to the other regions we analyzed [28]. In the brain regions where we observed a sharply decreased expression of neuron-specific genes we also detected a significant enrichment for those genes to reside in neuronal and synaptic process defined pathways. We also found a strong downregulation of neuron-specific genes in the IFG. Of note, the IFG is generally considered to be a brain region affected relatively late in AD [28]. Interestingly, the set of neuron-specific genes downregulated in the IFG are different compared to the other regions and are more closely enriched for “GPCR signaling” (G protein-coupled receptors) based on GSEA. GPCRs are implicated in AD and the processing of APP [29].

### Microglia show different patterns of expression across brain regions

Microglia specific genes demonstrate a consistent increased gene expression pattern across the brain regions we examined, except the FP. The discordant results observed for FP might be a consequence of the low number of microglia genes analyzed (*n* = 7). Our findings are concordant with the snRNA-Seq study describing upregulation of microglia genes [5]. Enrichment analysis demonstrated a prevalence of transcripts related to immune system processes across all of the regions, excluding FP and DLPFC. Among the specific immune processes detected in microglia specific genes, we observed “Neutrophil degranulation” and several classes of Toll-Like receptors (TLR) signaling cascades. These results confirm the known involvement of microglia in the brain immune processes [30] and the association with neuroinflammation observed in AD [6]. Multiple lines of evidence suggest that AD is promoted by innate immune mechanisms in the central nervous system [31, 32]

The role of neutrophils in AD has been reported in both animal models and humans. For example, it was observed in two transgenic models of AD (5xFAD and 3xTg-AD mice) that neutrophils infiltrate and accumulate in the CNS during all stages of AD and may play a role in microglia activation, synaptic dysfunction, and the accumulation of abnormal Aβ and tau [33]. Interestingly, astrocytes can differentially regulate neutrophil function, including degranulation [34], as well as produce neutrophil attractant chemokines [35]. This mechanism has not been described in microglia. However, both microglia and astrocytes in AD secrete pro-inflammatory cytokines into the surrounding brain tissue rich in Aβ deposits, potentially contributing to the intraparenchymal NET formation and generating crosstalk with intraparenchymal neutrophils [36]

### Endothelial genes are upregulated in AD and enriched for angiogenesis and vascular functional processes

Endothelial cell-specific transcripts were upregulated across all studied regions, with the exclusion of FP and IFG, the regions with the lowest number of DEGs. We observed a general enrichment of pathways related to vascular development and angiogenesis in the significant endothelial cell DEGs, especially in the TCX and CBE, but not in other regions. Vascular abnormalities are considered an essential hallmark of AD, and according to the “Vascular Hypothesis,” cerebral hypoperfusion may be a major contributor to AD pathogenesis [21]. Our results are also concordant with a recent study conducted in the Tg4510 tauopathy rodent model, where the authors observed increased brain vascularization, and an upregulation of vascular remodeling genes in endothelial cells of the transgenic animal [37]. Another study described the presence of upregulation of an angiogenic transcription factor (Ets-1) as well as its co-localization with VEGF in the AD brain [38].

### Astrocyte genes are enriched in SLC transport and immune processes in AD

Astrocyte genes were upregulated in 4 regions, in agreement with the snRNA-Seq study [5]. We were not able to detect many specific processes related to astrocytes in this study. Most of the genes were included in the non-specific “Developmental processes” category. Using REACTOME, we found enrichment for “SLC-mediated transmembrane transport” whereas using GSEA we detected enrichment in “Immune system” functional classes in FP and PHG, but they only included four and five genes, respectively. However, this last result would confirm a previous study conducted on astrocytes isolated by LCM from human AD brains [3].

### Oligodendrocytes are enriched for the Glycoprotein metabolism in Alzheimer’s disease

Oligodendrocytes were upregulated in almost all of the brain regions, as described in the snRNA-seq study [5], except for STG and CBE. We detected an enrichment of upregulated genes associated with post-synaptic membranes and proteoglycan metabolism. Proteoglycans, specifically glycosaminoglycans, play several roles in amyloid formation including promoting the aggregation of Aβ into insoluble amyloid fibrils, which contributes to the increased neurotoxicity of Aβ [39].

The role of oligodendrocytes in AD became relevant in recent years due to neuroimaging studies that highlighted the micro- and macro-structural abnormalities in white matter associated with disease progression [40]. The anomalies were primarily observed in areas with physiologically low perfusion levels and where white matter density is known to decrease in healthy aging and AD [41]. Interestingly, some studies reported that white matter pathology might emerge before the appearance of cortical plaques and tangles [42, 43], suggesting that white matter abnormalities, as well as impaired myelination and oligodendrocyte function, could promote cognitive impairment and AD pathology [40].

### Comparison with previous studies

Itoh et al. [13] conducted a study using public datasets from Parkinson’s Disease, Multiple Sclerosis and AD, classifying the genes using sorted cell data from the same database we used [44]. However, they did not define any specific cutoff to attribute the single cell expression, simply including the top 500 genes for each cell class. Additionally, they analyzed a small AD dataset (AD = 21; non-demented: ND = 22) from STG characterized with microarray technology, compared to our dataset which was characterized with RNA sequencing including 1,019 samples from 7 distinct brain regions. The results differ since they were not able to detect upregulation of endothelial and astrocytes genes in AD. Wang et al. [14] classified the DEGs obtained in their AD study across 19 brain regions using a method based on Bayesian negative binomial regression. Despite the large number of brain regions the sample size was limited (averaging 55 donors for each brain region). They found downregulation of neuronal genes across multiple regions, upregulation of neuronal genes in Inferior Frontal Gyrus (IFG), and upregulation of astrocyte and oligodendrocyte genes across a few regions. However, they were not able to find specific patterns for endothelial cells and they did not investigate cell-specific biological processes or pathways.

## CONCLUSIONS

In this manuscript, we describe a bioinformatics approach, termed ESHRD, that can leverage existing or new homogenate-based (“bulk”) RNA sequencing data to deconvolute and quantitate cell-specific transcripts from brain tissue. ESHRD can reliably detect cell-specific changes based on our LCM study of oligodendrocyte gene changes in multiple system atrophy patients. The ESHRD approach replicates previously published findings in neurons from AD patient brain specimens, and we extended our work to characterize novel AD-related changes in relatively unexplored cell types in AD, oligodendrocytes and endothelial cells.

The use of a simple bioinformatics-based approach to perform cell-specific analyses from homogenate data is not without limitations. The majority of transcripts in brain tissue is not cell-type specific and therefore cannot be assigned using ESHRD. Also, the alteration of a multi cell-specific transcript in only one particular cell type will not be identified by using our approach. For example, if a specific gene is part of basal cellular metabolism and was therefore classified as a “mixed” gene due to its expression in many cell types in the brain, if the presence of disease induces a change in this gene but only in one cell type ESHRD will not be able to assign that change appropriately. Additionally, our approach cannot identify a transcriptional effect whereby a microglial cell is influenced by the disease to begin transcribing a neuronal specific gene. In that case, ESHRD would assign the changes in the neuronal gene transcription to neurons and not to microglia where in reality the underlying transcriptional changes are occurring.

Despite these limitations, we propose that the approach described herein is an extremely beneficial way to leverage existing bulk RNA-Seq data and may constitute a “first step” toward cell-specific investigations by many who are not able to perform LCM or scRNA-Seq on their samples. Large-sized studies are essential to capture information about human variation in disease and while cell-specific transcriptional analyses are possible via LCM or scRNA-Seq their associated time and cost restraints typically force the execution of a study design that consists of a smaller collection of cases and controls. We estimate that the costs associated with LCM or scRNA-Seq are approximately 5X that of a bulk tissue sequencing study, therefore, for the same cost one may conduct a cell-type specific experiment that is dramatically underpowered compared to a bulk tissue study. Additionally, using the ESHRD deconvolution approach one can garner information on many cell types at once; however, with LCM, this goal would be a multiplier of effort and costs. The use of scRNA-Seq is revolutionizing how transcriptional profiling is performed. However, one of the major constraints on the scRNA-Seq workflow is the creation of reproducible single cell suspensions from brain tissue and in fact most studies in humans utilize single nuclei sequencing as a single cell suspension of intact whole cells is difficult to obtain from frozen tissue. While scRNA-Seq can indeed provide an unbiased profile of all cell types in the sample that is only true if the complications associated with the creation of the uniform single cell or nuclei suspension can be avoided. There are no such concerns with homogenate-based RNA sequencing since the RNA is isolated in bulk from a piece of dissected brain tissue. In short, we provide an analytical approach that we demonstrate is informative and cost/time effective for extracting cell-type specific information from brain homogenate RNA profiling data.

## MATERIALS AND METHODS

### ESHRD score and gene classification

We downloaded the data from Zhang et al. [44], including the cell-specific expression values determined by RNA sequencing from mouse cerebral cortex, (http://web.stanford.edu/group/barres_lab/brain_rnaseq.html). The dataset consists of expression values in Fragments Per Kilobase transcript per Million mapped reads (*fpkm*) for 22,458 genes in: neurons (N), astrocytes (A), microglia (M), myelinating oligodendrocytes (MO), newly formed oligodendrocytes (NFO), oligodendrocyte precursor cells (OPC), and endothelial cells (EC). The original authors noted that the OPC fraction is contaminated with 5% of microglia [44]. We removed genes with *fpkm* = 0.1 in all of the cell types (*n* = 6,157 genes removed) because this level of expression is deficient and may contribute false positive noise to the assessment of cell-specific transcripts. Therefore, the final dataset used for our classification approach included 16,301 genes. After the conversion of the mouse gene symbols to known human genes, the definitive database used for classification contained 13,384 unique *H. sapiens* Ensembl IDs.

For each gene and cell type, we computed an enrichment score (ES) by dividing the *fpkm* value in the reference cell type by the averaged *fpkm* values from the other cell types. Then, each gene was assigned to one or multiple cell classes according to their ES. First, we computed the highest ES (ES_high_) for each gene. Then, comparing the ES_high_ with the other scores for each cell type, we defined three different classes of genes: a) cell-specific; b) multiple cell-specific, and c) mixed. Class “*a*” (cell-specific) included genes highly expressed only in one cell type, and with demonstrably low or no expression in all the other cells. Specifically, the ES in the other cells was less than or equal to 25% of the ES_high_ (ES ≤ ES_high_ x 0.25). Class “*b*” (multiple cell-specific) includes genes simultaneously highly expressed in more than one cell type. For this gene class, ES should be greater than or equal to 75% of the ES_high_ (ES ≥ ES_high_ x 0.75). Finally, class “*c*” (mixed) includes genes with at least one cell type with ES between 25% and 75% of ES_high_. For this class of genes, it is impossible to assign the gene to only a small set of cell types in the brain. Specifically for this class (ES_high_ x 0.75) ≥ ES ≥ (ES_high_ x 0.25). We also created an additional pan-oligodendrocyte class by combining across all of the oligodendrocyte sub-types (MO, NFO, OPC, MO/NFO, MO/OPC, and NFO/OPC). We term this bioinformatics approach Enrichment Score Homogenate RNA Deconvolution or “ESHRD” (pronounced e-shred).

The enrichment for specific cell types was conducted by permutation analysis. In each differential expression dataset, and for each cell type, we randomly sampled (50,000 times) from the entire list of genes the number of genes corresponding to the DEGs detected for that specific cell type. For each permutation, we assessed the number of genes expressed in that particular cell type, and we compared that to the actual number of DEGs detected. We considered it to be a statistically significant enrichment when the number of observed cell-specific DEGs was larger than the number of cell-specific sampled genes in at least 95% of the permutations.

### Multiple system atrophy dataset

We used an MSA dataset to conduct a comparative analysis between brain homogenates, and LCM’d oligodendrocytes from the same donors following expression profiling. We performed RNA profiling on white matter cerebellum samples from 4 MSA patients and five controls, both on tissue homogenates and oligodendrocytes isolated by LCM. Samples were from the Brain and Body Donation Program (Sun City, AZ) [45] and New South Wales (NSW) Brain Bank (Sydney, AU). Sex, age, and PMI distribution between cases and controls were not significantly different (*p* > 0.200). For the brain homogenate profiling, RNA was extracted using the Qiagen miRNAeasy kit and was DNAse-treated (Qiagen). Quality was assessed by Bioanalyzer (Agilent). Sequencing libraries were prepared with 250 ng of total RNA using Illumina’s Truseq RNA Sample Preparation Kit v2 (Illumina, Inc.) following the manufacturer’s protocol. The final library was sequenced by 50 bp paired-end sequencing on a HiSeq 2500.

For the isolation of oligodendrocytes, a total of 300 cells per sample were captured using Arcturus CapSure Macro LCM Caps (Applied Biosystems). Oligodendrocytes were identified using a modified H&E staining protocol adapted from Ordway et al. [46]. RNA was extracted immediately after cell capture using the Arcturus PicoPure RNA Isolation Kit (Applied Biosystems). For library preparation, the SMARTer® Stranded Total RNA-Seq Kit - Pico Input (Clontech/Takara) was used. Samples were sequenced (2 x 75 bp paired-end run) on the Illumina HiSeq2500.

Reads were aligned to the Human reference genome (GRCh37) using the Spliced Transcripts Alignment to a Reference (STAR) software v2.5 [47], then summarized as gene-level counts using featureCounts 1.4.4 [48]. Outlier and batch effect detection were conducted through Principal Component Analysis (PCA), using R software v3.3.1 [49]. Gene expression differential analyses between MSA cases and controls were conducted using the R package DESeq2 v1.14.1 [50] including age, gender, PMI and sample source as covariates. The p-values were adjusted using the False Discovery Rate (FDR) method [51].

### Alzheimer’s disease datasets

We applied our ESHRD method to different human RNA sequencing datasets from Late Onset AD patients and ND (non-demented) control samples. We considered: temporal cortex (TCX), superior temporal gyrus (STG), dorsolateral prefrontal cortex (DLPFC), frontal pole (FP), inferior frontal gyrus (IFG), parahippocampal gyrus (PHG), and cerebellum (CBE). TCX and CBE data were from the Mayo Study [52], DLPFC data were from ROSMAP study (Religious Order Study and Memory and Aging Project) [53], and STG, FP, IFG, and PHG were from the Mount Sinai study [54] (AD = 633; ND = 383) (Table 2).

**Table 2:** Sample size and details of the datasets used in this study

**Brain Region**	**AD**	**ND**	**Study**
Temporal Cortex (TCX)	80	71	MAYO
Superior Temporal Gyrus (STG)	85	37	MOUNT SINAI
Dorsolateral Prefrontal Cortex (DLPFC)	155	86	ROSMAP
Frontal Pole (FP)	90	45	MOUNT SINAI
Inferior Frontal Gyrus (IFG)	79	37	MOUNT SINAI
Parahippocampal Gyrus (PHG)	65	38	MOUNT SINAI
Cerebellum (CBE)	79	72	MAYO

Differential expression results were downloaded from the AMP-AD portal (#syn14237651) selecting the “Diagnosis” model, where the AD and control definitions were harmonized across studies by defining cognitive scores, Braak staging, and tau pathology. All the details of the analysis workflow can be found at https://www.synapse.org/#!Synapse:syn14237651. Briefly, library normalization and covariate adjustment were conducted for each study separately. Low expressed genes showing less than 1 CPM (read Counts Per Million Total reads) in at least 50% of samples in each tissue and diagnosis category were filtered out. Data were normalized with conditional quantile normalization and then using a weighted linear model using *voom-limma* package [55]. Outliers were removed if both identified by hierarchical clustering and PCA. Expression values were adjusted for covariates associated with top principal components explaining more than 1% of variance of expression residuals. Differential expression was performed as weighted fixed/mixed effect linear models using the *voom-limma* package in R separately for each study. For the Mayo and MSMM study, including multiple brain regions, donor specific effects were explicitly modeled as random effects. For the ESHRD classification, we selected differentially expressed genes (DEGs) with FDR < 0.05.

### Pathway and enrichment analysis

Enrichment analysis was performed with Gene Ontology (GO) [56] and REACTOME [57] databases. We also conducted Gene Set Enrichment Analysis (GSEA), referencing to the REACTOME database using 10,000 permutations. In this case, we used all of the genes classified as the input list, regardless of their FDR. All of the analyses were conducted using the R packages *anRichmentMethods* and *ReactomePA* [58]. The p-values were adjusted with the Bonferroni method and pathways were considered significant at adj *p* < 0.05.

## Supplementary Material

Supplementary Figures

Supplementary Table 1

Supplementary Table 2

Supplementary Table 3

Supplementary Table 4

Supplementary Table 5

Supplementary Table 6

## References

[1] Mastroeni D, Sekar S, Nolz J, Delvaux E, Lunnon K, Mill J, Liang WS, Coleman PD. ANK1 is up-regulated in laser captured microglia in Alzheimer’s brain; the importance of addressing cellular heterogeneity. PLoS One. 2017; 12:e0177814. 10.1371/journal.pone.0177814 28700589 PMC5507536

[2] Mastroeni D, Nolz J, Sekar S, Delvaux E, Serrano G, Cuyugan L, Liang WS, Beach TG, Rogers J, Coleman PD. Laser-captured microglia in the Alzheimer’s and Parkinson’s brain reveal unique regional expression profiles and suggest a potential role for hepatitis B in the Alzheimer’s brain. Neurobiol Aging. 2018; 63:12–21. 10.1016/j.neurobiolaging.2017.10.019 29207277 PMC6686891

[3] Sekar S, McDonald J, Cuyugan L, Aldrich J, Kurdoglu A, Adkins J, Serrano G, Beach TG, Craig DW, Valla J, Reiman EM, Liang WS. Alzheimer’s disease is associated with altered expression of genes involved in immune response and mitochondrial processes in astrocytes. Neurobiol Aging. 2015; 36:583–91. 10.1016/j.neurobiolaging.2014.09.027 25448601 PMC4315763

[4] Liang WS, Dunckley T, Beach TG, Grover A, Mastroeni D, Ramsey K, Caselli RJ, Kukull WA, McKeel D, Morris JC, Hulette CM, Schmechel D, Reiman EM, et al. Altered neuronal gene expression in brain regions differentially affected by Alzheimer’s disease: a reference data set. Physiol Genomics. 2008; 33:240–56. 10.1152/physiolgenomics.00242.2007 18270320 PMC2826117

[5] Mathys H, Davila-Velderrain J, Peng Z, Gao F, Mohammadi S, Young JZ, Menon M, He L, Abdurrob F, Jiang X, Martorell AJ, Ransohoff RM, Hafler BP, et al. Single-cell transcriptomic analysis of Alzheimer’s disease. Nature. 2019; 570:332–37. 10.1038/s41586-019-1195-2 31042697 PMC6865822

[6] Selles MC, Oliveira MM, Ferreira ST. Brain Inflammation Connects Cognitive and Non-Cognitive Symptoms in Alzheimer’s Disease. J Alzheimers Dis. 2018; 64:S313–27. 10.3233/JAD-179925 29710716

[7] González-Reyes RE, Nava-Mesa MO, Vargas-Sánchez K, Ariza-Salamanca D, Mora-Muñoz L. Involvement of Astrocytes in Alzheimer’s Disease from a Neuroinflammatory and Oxidative Stress Perspective. Front Mol Neurosci. 2017; 10:427. 10.3389/fnmol.2017.00427 29311817 PMC5742194

[8] Hollands C, Bartolotti N, Lazarov O. Alzheimer’s Disease and Hippocampal Adult Neurogenesis; Exploring Shared Mechanisms. Front Neurosci. 2016; 10:178. 10.3389/fnins.2016.00178 27199641 PMC4853383

[9] Sun X, He G, Qing H, Zhou W, Dobie F, Cai F, Staufenbiel M, Huang LE, Song W. Hypoxia facilitates Alzheimer’s disease pathogenesis by up-regulating BACE1 gene expression. Proc Natl Acad Sci USA. 2006; 103:18727–32. 10.1073/pnas.0606298103 17121991 PMC1693730

[10] Kelleher RJ, Soiza RL. Evidence of endothelial dysfunction in the development of Alzheimer’s disease: is Alzheimer’s a vascular disorder? Am J Cardiovasc Dis. 2013; 3:197–226. 24224133 PMC3819581

[11] Iadecola C. The pathobiology of vascular dementia. Neuron. 2013; 80:844–66. 10.1016/j.neuron.2013.10.008 24267647 PMC3842016

[12] Tse KH, Herrup K. DNA damage in the oligodendrocyte lineage and its role in brain aging. Mech Ageing Dev. 2017; 161:37–50. 10.1016/j.mad.2016.05.006 27235538 PMC5124419

[13] Itoh Y, Voskuhl RR. Cell specificity dictates similarities in gene expression in multiple sclerosis, Parkinson’s disease, and Alzheimer’s disease. PLoS One. 2017; 12:e0181349. 10.1371/journal.pone.0181349 28715462 PMC5513529

[14] Wang M, Roussos P, McKenzie A, Zhou X, Kajiwara Y, Brennand KJ, De Luca GC, Crary JF, Casaccia P, Buxbaum JD, Ehrlich M, Gandy S, Goate A, et al. Integrative network analysis of nineteen brain regions identifies molecular signatures and networks underlying selective regional vulnerability to Alzheimer’s disease. Genome Med. 2016; 8:104. 10.1186/s13073-016-0355-3 27799057 PMC5088659

[15] Ullian EM, Sapperstein SK, Christopherson KS, Barres BA. Control of synapse number by glia. Science. 2001; 291:657–61. 10.1126/science.291.5504.657 11158678

[16] Doetsch F. The glial identity of neural stem cells. Nat Neurosci. 2003; 6:1127–34. 10.1038/nn1144 14583753

[17] Noctor SC, Martínez-Cerdeño V, Kriegstein AR. Contribution of intermediate progenitor cells to cortical histogenesis. Arch Neurol. 2007; 64:639–42. 10.1001/archneur.64.5.639 17502462

[18] Azevedo FA, Carvalho LR, Grinberg LT, Farfel JM, Ferretti RE, Leite RE, Jacob Filho W, Lent R, Herculano-Houzel S. Equal numbers of neuronal and nonneuronal cells make the human brain an isometrically scaled-up primate brain. J Comp Neurol. 2009; 513:532–41. 10.1002/cne.21974 19226510

[19] Crews L, Masliah E. Molecular mechanisms of neurodegeneration in Alzheimer’s disease. Hum Mol Genet. 2010; 19:R12–20. 10.1093/hmg/ddq160 20413653 PMC2875049

[20] Calsolaro V, Edison P. Neuroinflammation in Alzheimer’s disease: current evidence and future directions. Alzheimers Dement. 2016; 12:719–32. 10.1016/j.jalz.2016.02.010 27179961

[21] de la Torre JC. The vascular hypothesis of Alzheimer’s disease: bench to bedside and beyond. Neurodegener Dis. 2010; 7:116–21. 10.1159/000285520 20173340

[22] Tamagnini F, Scullion S, Brown JT, Randall AD. Intrinsic excitability changes induced by acute treatment of hippocampal CA1 pyramidal neurons with exogenous amyloid β peptide. Hippocampus. 2015; 25:786–97. 10.1002/hipo.22403 25515596 PMC4791149

[23] Ren SC, Chen PZ, Jiang HH, Mi Z, Xu F, Hu B, Zhang J, Zhu ZR. Persistent sodium currents contribute to Aβ1-42-induced hyperexcitation of hippocampal CA1 pyramidal neurons. Neurosci Lett. 2014; 580:62–67. 10.1016/j.neulet.2014.07.050 25102326

[24] Busche MA, Chen X, Henning HA, Reichwald J, Staufenbiel M, Sakmann B, Konnerth A. Critical role of soluble amyloid-β for early hippocampal hyperactivity in a mouse model of Alzheimer’s disease. Proc Natl Acad Sci USA. 2012; 109:8740–45. 10.1073/pnas.1206171109 22592800 PMC3365221

[25] Leonard AS, McNamara JO. Does epileptiform activity contribute to cognitive impairment in Alzheimer’s disease? Neuron. 2007; 55:677–78. 10.1016/j.neuron.2007.08.014 17785172

[26] Bakker A, Krauss GL, Albert MS, Speck CL, Jones LR, Stark CE, Yassa MA, Bassett SS, Shelton AL, Gallagher M. Reduction of hippocampal hyperactivity improves cognition in amnestic mild cognitive impairment. Neuron. 2012; 74:467–74. 10.1016/j.neuron.2012.03.023 22578498 PMC3351697

[27] Ciccone R, Franco C, Piccialli I, Boscia F, Casamassa A, de Rosa V, Cepparulo P, Cataldi M, Annunziato L, Pannaccione A. Amyloid β-Induced Upregulation of Na_v_1.6 Underlies Neuronal Hyperactivity in Tg2576 Alzheimer’s Disease Mouse Model. Sci Rep. 2019; 9:13592. 10.1038/s41598-019-50018-1 31537873 PMC6753212

[28] Ball MJ, Delacourte A. The biochemical pathway of neurofibrillary degeneration in aging and Alzheimer’s disease. Neurology. 2000; 54:538. 10.1212/WNL.54.2.538 10668745

[29] Zhao J, Deng Y, Jiang Z, Qing H. G protein-coupled receptors (GPCRs) in Alzheimer’s disease: A focus on BACE1 related GPCRs. Front Aging Neurosci. 2016; 8:58. 10.3389/fnagi.2016.00058 27047374 PMC4805599

[30] Hanisch UK, Kettenmann H. Microglia: active sensor and versatile effector cells in the normal and pathologic brain. Nat Neurosci. 2007; 10:1387–94. 10.1038/nn1997 17965659

[31] Wyss-Coray T. Inflammation in Alzheimer disease: driving force, bystander or beneficial response? Nat Med. 2006; 12:1005–15. 10.1038/nm1484 16960575

[32] Schwartz M, Kipnis J, Rivest S, Prat A. How do immune cells support and shape the brain in health, disease, and aging? J Neurosci. 2013; 33:17587–96. 10.1523/JNEUROSCI.3241-13.2013 24198349 PMC3818540

[33] Zenaro E, Pietronigro E, Della Bianca V, Piacentino G, Marongiu L, Budui S, Turano E, Rossi B, Angiari S, Dusi S, Montresor A, Carlucci T, Nanì S, et al. Neutrophils promote Alzheimer’s disease-like pathology and cognitive decline via LFA-1 integrin. Nat Med. 2015; 21:880–86. 10.1038/nm.3913 26214837

[34] Xie L, Poteet EC, Li W, Scott AE, Liu R, Wen Y, Ghorpade A, Simpkins JW, Yang SH. Modulation of polymorphonuclear neutrophil functions by astrocytes. J Neuroinflammation. 2010; 7:53. 10.1186/1742-2094-7-53 20828397 PMC2942816

[35] Pereira CF, Middel J, Jansen G, Verhoef J, Nottet HS. Enhanced expression of fractalkine in HIV-1 associated dementia. J Neuroimmunol. 2001; 115:168–75. 10.1016/S0165-5728(01)00262-4 11282167

[36] Pietronigro EC, Della Bianca V, Zenaro E, Constantin G. NETosis in Alzheimer’s disease. Front Immunol. 2017; 8:211. 10.3389/fimmu.2017.00211 28303140 PMC5332471

[37] Bennett RE, Robbins AB, Hu M, Cao X, Betensky RA, Clark T, Das S, Hyman BT. Tau induces blood vessel abnormalities and angiogenesis-related gene expression in P301L transgenic mice and human Alzheimer’s disease. Proc Natl Acad Sci USA. 2018; 115:E1289–98. 10.1073/pnas.1710329115 29358399 PMC5819390

[38] Jantaratnotai N, Ling A, Cheng J, Schwab C, McGeer PL, McLarnon JG. Upregulation and expression patterns of the angiogenic transcription factor ets-1 in Alzheimer’s disease brain. J Alzheimers Dis. 2013; 37:367–77. 10.3233/JAD-122191 23948889

[39] Ariga T, Miyatake T, Yu RK. Role of proteoglycans and glycosaminoglycans in the pathogenesis of Alzheimer’s disease and related disorders: amyloidogenesis and therapeutic strategies—a review. J Neurosci Res. 2010; 88:2303–15. 10.1002/jnr.22393 20623617

[40] Nasrabady SE, Rizvi B, Goldman JE, Brickman AM. White matter changes in Alzheimer’s disease: a focus on myelin and oligodendrocytes. Acta Neuropathol Commun. 2018; 6:22. 10.1186/s40478-018-0515-3 29499767 PMC5834839

[41] Brown WR, Thore CR. Review: cerebral microvascular pathology in ageing and neurodegeneration. Neuropathol Appl Neurobiol. 2011; 37:56–74. 10.1111/j.1365-2990.2010.01139.x 20946471 PMC3020267

[42] Desai MK, Mastrangelo MA, Ryan DA, Sudol KL, Narrow WC, Bowers WJ. Early oligodendrocyte/myelin pathology in Alzheimer’s disease mice constitutes a novel therapeutic target. Am J Pathol. 2010; 177:1422–35. 10.2353/ajpath.2010.100087 20696774 PMC2928974

[43] Desai MK, Sudol KL, Janelsins MC, Mastrangelo MA, Frazer ME, Bowers WJ. Triple-transgenic Alzheimer’s disease mice exhibit region-specific abnormalities in brain myelination patterns prior to appearance of amyloid and tau pathology. Glia. 2009; 57:54–65. 10.1002/glia.20734 18661556 PMC2584762

[44] Zhang Y, Chen K, Sloan SA, Bennett ML, Scholze AR, O’Keeffe S, Phatnani HP, Guarnieri P, Caneda C, Ruderisch N, Deng S, Liddelow SA, Zhang C, et al. An RNA-sequencing transcriptome and splicing database of glia, neurons, and vascular cells of the cerebral cortex. J Neurosci. 2014; 34:11929–47. 10.1523/JNEUROSCI.1860-14.2014 25186741 PMC4152602

[45] Beach TG, Adler CH, Sue LI, Serrano G, Shill HA, Walker DG, Lue L, Roher AE, Dugger BN, Maarouf C, Birdsill AC, Intorcia A, Saxon-Labelle M, et al. Arizona Study of Aging and Neurodegenerative Disorders and Brain and Body Donation Program. Neuropathology. 2015; 35:354–89. 10.1111/neup.12189 25619230 PMC4593391

[46] Ordway GA, Szebeni A, Duffourc MM, Dessus-Babus S, Szebeni K. Gene expression analyses of neurons, astrocytes, and oligodendrocytes isolated by laser capture microdissection from human brain: detrimental effects of laboratory humidity. J Neurosci Res. 2009; 87:2430–38. 10.1002/jnr.22078 19360883 PMC2922841

[47] Dobin A, Davis CA, Schlesinger F, Drenkow J, Zaleski C, Jha S, Batut P, Chaisson M, Gingeras TR. STAR: ultrafast universal RNA-seq aligner. Bioinformatics. 2013; 29:15–21. 10.1093/bioinformatics/bts635 23104886 PMC3530905

[48] Liao Y, Smyth GK, Shi W. featureCounts: an efficient general purpose program for assigning sequence reads to genomic features. Bioinformatics. 2014; 30:923–30. 10.1093/bioinformatics/btt656 24227677

[49] R Core Team. R Development Core Team. R: A Language and Environment for Statistical Computing. 2016 p. 275–86. Available from: https://www.r-project.org/

[50] Love MI, Huber W, Anders S. Moderated estimation of fold change and dispersion for RNA-seq data with DESeq2. Genome Biol. 2014; 15:550. 10.1186/s13059-014-0550-8 25516281 PMC4302049

[51] Benjamini Y, Hochberg Y, Benjamini Y, Hochberg Y. Controlling the false discovery rate: a practical and powerful approach to multiple testing. J R Stat Soc B. 1995; 57:289–300. 10.1111/j.2517-6161.1995.tb02031.x

[52] Allen M, Carrasquillo MM, Funk C, Heavner BD, Zou F, Younkin CS, Burgess JD, Chai HS, Crook J, Eddy JA, Li H, Logsdon B, Peters MA, et al. Human whole genome genotype and transcriptome data for Alzheimer’s and other neurodegenerative diseases. Sci Data. 2016; 3:160089. 10.1038/sdata.2016.89 27727239 PMC5058336

[53] Bennett DA, Schneider JA, Arvanitakis Z, Wilson RS. Overview and findings from the religious orders study. Curr Alzheimer Res. 2012; 9:628–45. 10.2174/156720512801322573 22471860 PMC3409291

[54] Wang M, Beckmann ND, Roussos P, Wang E, Zhou X, Wang Q, Ming C, Neff R, Ma W, Fullard JF, Hauberg ME, Bendl J, Peters MA, et al. The Mount Sinai cohort of large-scale genomic, transcriptomic and proteomic data in Alzheimer’s disease. Sci Data. 2018; 5:180185. 10.1038/sdata.2018.185 30204156 PMC6132187

[55] Law CW, Chen Y, Shi W, Smyth GK. voom: precision weights unlock linear model analysis tools for RNA-seq read counts. Genome Biol. 2014; 15:R29. 10.1186/gb-2014-15-2-r29 24485249 PMC4053721

[56] Ashburner M, Ball CA, Blake JA, Botstein D, Butler H, Cherry JM, Davis AP, Dolinski K, Dwight SS, Eppig JT, Harris MA, Hill DP, Issel-Tarver L, et al, and The Gene Ontology Consortium. Gene ontology: tool for the unification of biology. Nat Genet. 2000; 25:25–29. 10.1038/75556 10802651 PMC3037419

[57] Fabregat A, Sidiropoulos K, Garapati P, Gillespie M, Hausmann K, Haw R, Jassal B, Jupe S, Korninger F, McKay S, Matthews L, May B, Milacic M, et al. The reactome pathway knowledgebase. Nucleic Acids Res. 2016; 44:D481–87. 10.1093/nar/gkv1351 26656494 PMC4702931

[58] Yu G, He QY. ReactomePA: an R/Bioconductor package for reactome pathway analysis and visualization. Mol Biosyst. 2016; 12:477–79. 10.1039/C5MB00663E 26661513

