# Supplementary material for: ESHRD: deconvolution of brain homogenate RNA expression data to identify cell-type-specific alterations in Alzheimer’s disease
Source: Aging (Albany NY). 2020 Mar 2;12(5):4124–62. doi: 10.18632/aging.102840 (PMC7093163; doi:10.18632/aging.102840)
Supplement: Supplementary Figures [file aging-12-102840-s007..pdf]

SUPPLEMENTARY FIGURES

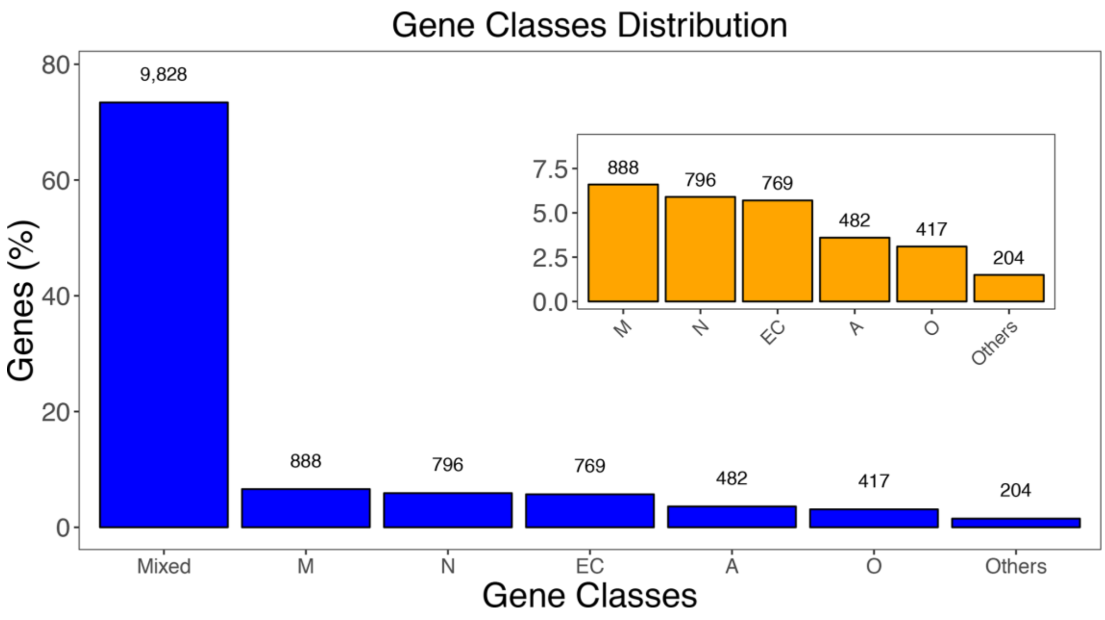

Supplementary Figure 1. Barplot representing the number of genes for each class in the Single Cell RNA sequencing database [10].

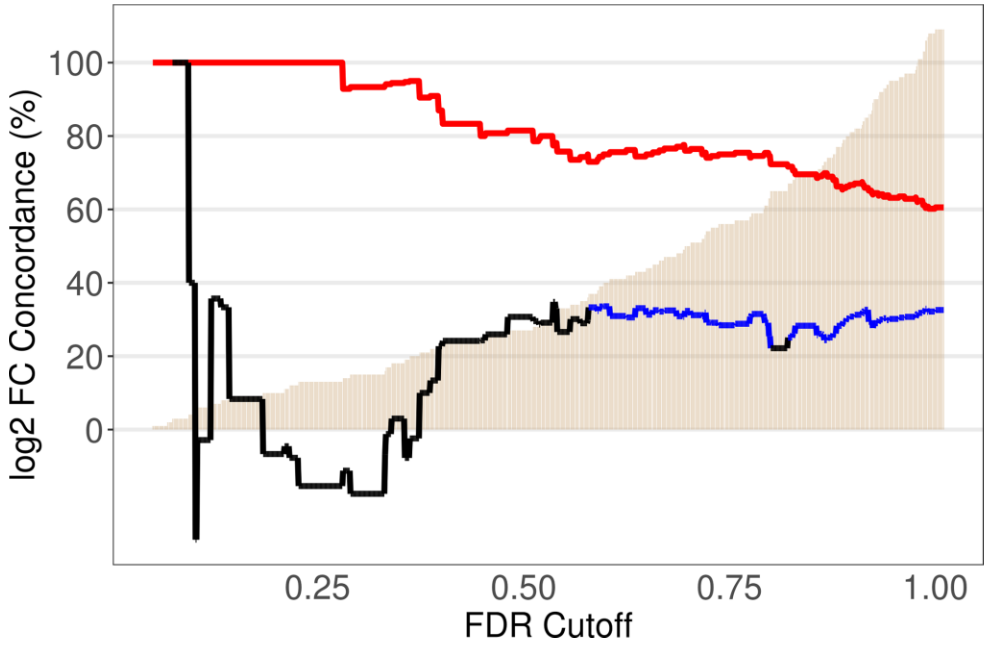

Supplementary Figure 2. Comparison of expression profiling of oligodendrocyte genes between bulk tissue and oligodendrocytes isolated by LCM in the same samples. The red line is the percent concordance of the direction of effect between bulk RNA-Seq and LCM. The Black and Blue line reflects the Spearman correlation coefficient (multiplied by 100) for the log2 fold-change between the two groups – black represents non-significant correlation coefficient results and the blue line is for significant results. The bar plot in the background reflects the number of genes included in the analysis.

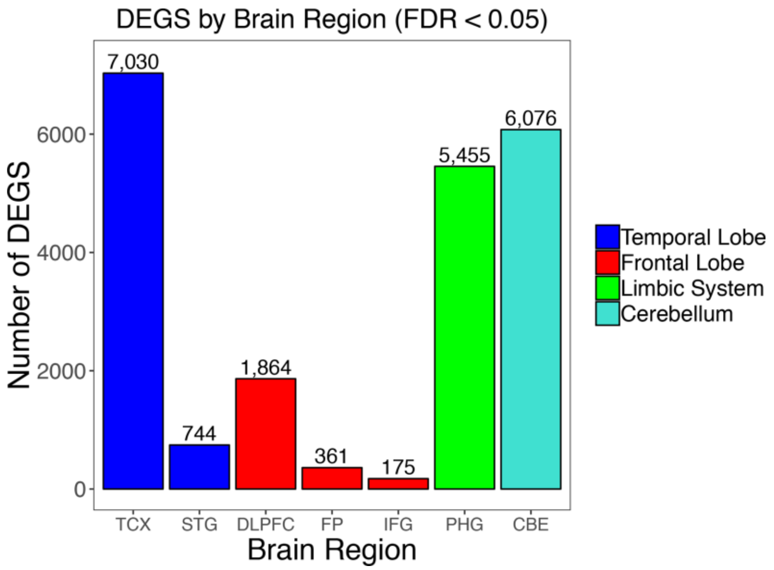

Supplementary Figure 3. Differentially expressed genes classified for the Alzheimer's Disease dataset. TCX: temporal cortex; STG: superior temporal gyrus; DLPFC: dorso-lateral prefrontal cortex; FP: frontal pole; IFG: inferior frontal gyrus; PHG: para-hippocampal gyrus; CBE: cerebellum.

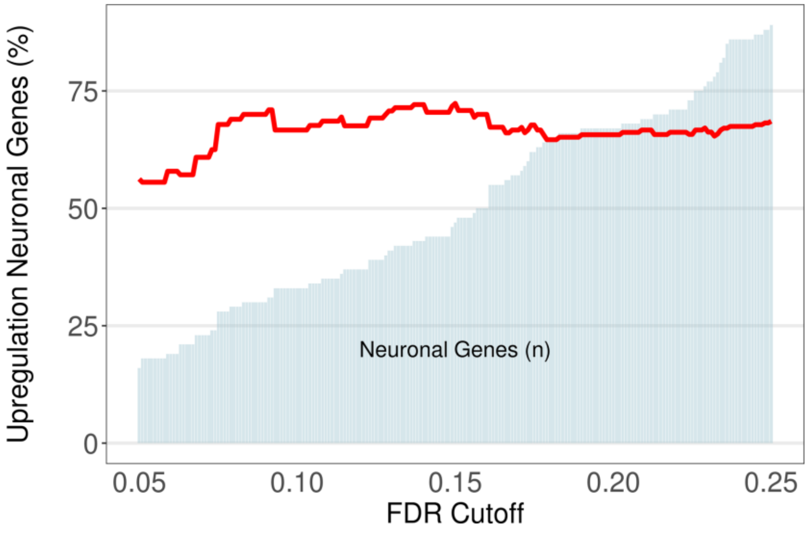

Supplementary Figure 4. Pattern of downregulation/upregulation for neuronal specific genes in FP as a function of different FDR cutoffs. The red line is the percent of upregulated neuronal genes in FP. The bar plot in the background reflects the number of genes included in the analysis depending on the FDR cutoff.

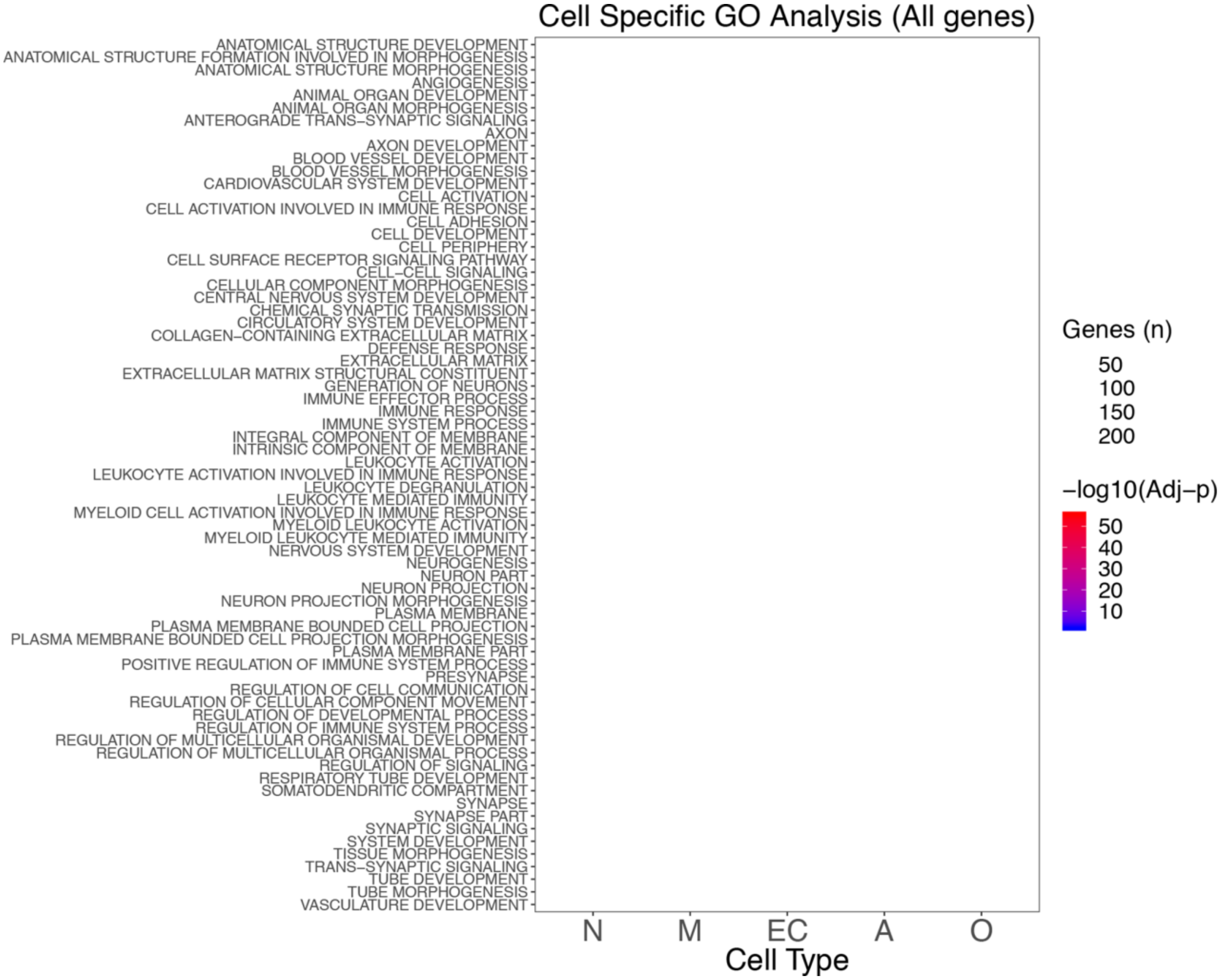
Cell Specific GO Analysis (All genes)
ANATOMICAL STRUCTURE DEVELOPMENT
ANATOMICAL STRUCTURE FORMATION INVOLVED IN MORPHOGENESIS
ANATOMICAL STRUCTURE MORPHOGENESIS
ANGIOGENESIS
ANIMAL ORGAN DEVELOPMENT
ANIMAL ORGAN MORPHOGENESIS
ANTEROGRADE TRANS–SYNAPTIC SIGNALING
AXON
AXON DEVELOPMENT
BLOOD VESSEL DEVELOPMENT
BLOOD VESSEL MORPHOGENESIS
CARDIOVASCULAR SYSTEM DEVELOPMENT
CELL ACTIVATION
CELL ACTIVATION INVOLVED IN IMMUNE RESPONSE
CELL ADHESION
CELL DEVELOPMENT
CELL PERIPHERY
CELL SURFACE RECEPTOR SIGNALING PATHWAY
CELL–CELL SIGNALING
CELLULAR COMPONENT MORPHOGENESIS
CENTRAL NERVOUS SYSTEM DEVELOPMENT
CHEMICAL SYNAPTIC TRANSMISSION
CIRCULATORY SYSTEM DEVELOPMENT
COLLAGEN–CONTAINING EXTRACELLULAR MATRIX
DEFENSE RESPONSE
EXTRACELLULAR MATRIX
EXTRACELLULAR MATRIX STRUCTURAL CONSTITUENT
GENERATION OF NEURONS
IMMUNE EFFECTOR PROCESS
IMMUNE RESPONSE
IMMUNE SYSTEM PROCESS
INTEGRAL COMPONENT OF MEMBRANE
INTRINSIC COMPONENT OF MEMBRANE
LEUKOCYTE ACTIVATION
LEUKOCYTE ACTIVATION INVOLVED IN IMMUNE RESPONSE
LEUKOCYTE DEGRANULATION
LEUKOCYTE MEDIATED IMMUNITY
MYELOID CELL ACTIVATION INVOLVED IN IMMUNE RESPONSE
MYELOID LEUKOCYTE ACTIVATION
MYELOID LEUKOCYTE MEDIATED IMMUNITY
NERVOUS SYSTEM DEVELOPMENT
NEUROGENESIS
NEURON PART
NEURON PROJECTION
NEURON PROJECTION MORPHOGENESIS
PLASMA MEMBRANE
PLASMA MEMBRANE BOUNDED CELL PROJECTION
PLASMA MEMBRANE BOUNDED CELL PROJECTION MORPHOGENESIS
PLASMA MEMBRANE PART
POSITIVE REGULATION OF IMMUNE SYSTEM PROCESS
PRESYNAPSE
REGULATION OF CELL COMMUNICATION
REGULATION OF CELLULAR COMPONENT MOVEMENT
REGULATION OF DEVELOPMENTAL PROCESS
REGULATION OF IMMUNE SYSTEM PROCESS
REGULATION OF MULTICELLULAR ORGANISMAL DEVELOPMENT
REGULATION OF MULTICELLULAR ORGANISMAL PROCESS
REGULATION OF SIGNALING
RESPIRATORY TUBE DEVELOPMENT
SOMATODENDRITIC COMPARTMENT
SYNAPSE
SYNAPSE PART
SYNAPTIC SIGNALING
SYSTEM DEVELOPMENT
TISSUE MORPHOGENESIS
TRANS–SYNAPTIC SIGNALING
TUBE DEVELOPMENT
TUBE MORPHOGENESIS
VASCULATURE DEVELOPMENT
N
M
EC
A
O
Cell Type
Genes (n)
50
100
150
200
−log10(Adj−p)
50
40
30
20
10

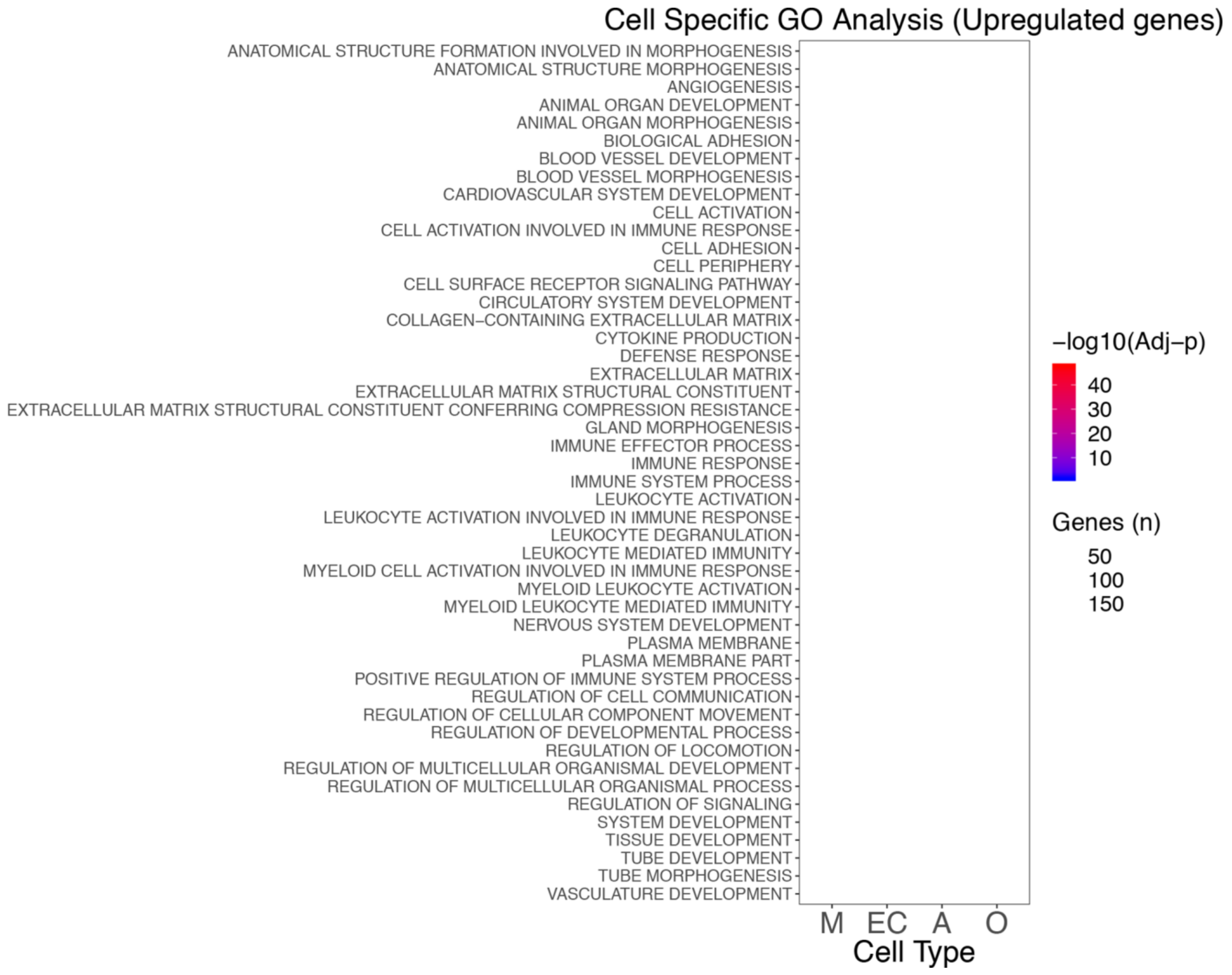
Cell Specific GO Analysis (Upregulated genes)
ANATOMICAL STRUCTURE FORMATION INVOLVED IN MORPHOGENESIS
ANATOMICAL STRUCTURE MORPHOGENESIS
ANGIOGENESIS
ANIMAL ORGAN DEVELOPMENT
ANIMAL ORGAN MORPHOGENESIS
BIOLOGICAL ADHESION
BLOOD VESSEL DEVELOPMENT
BLOOD VESSEL MORPHOGENESIS
CARDIOVASCULAR SYSTEM DEVELOPMENT
CELL ACTIVATION
CELL ACTIVATION INVOLVED IN IMMUNE RESPONSE
CELL ADHESION
CELL PERIPHERY
CELL SURFACE RECEPTOR SIGNALING PATHWAY
CIRCULATORY SYSTEM DEVELOPMENT
COLLAGEN−CONTAINING EXTRACELLULAR MATRIX
CYTOKINE PRODUCTION
DEFENSE RESPONSE
EXTRACELLULAR MATRIX
EXTRACELLULAR MATRIX STRUCTURAL CONSTITUENT
EXTRACELLULAR MATRIX STRUCTURAL CONSTITUENT CONFERRING COMPRESSION RESISTANCE
GLAND MORPHOGENESIS
IMMUNE EFFECTOR PROCESS
IMMUNE RESPONSE
IMMUNE SYSTEM PROCESS
LEUKOCYTE ACTIVATION
LEUKOCYTE ACTIVATION INVOLVED IN IMMUNE RESPONSE
LEUKOCYTE DEGRANULATION
LEUKOCYTE MEDIATED IMMUNITY
MYELOID CELL ACTIVATION INVOLVED IN IMMUNE RESPONSE
MYELOID LEUKOCYTE ACTIVATION
MYELOID LEUKOCYTE MEDIATED IMMUNITY
NERVOUS SYSTEM DEVELOPMENT
PLASMA MEMBRANE
PLASMA MEMBRANE PART
POSITIVE REGULATION OF IMMUNE SYSTEM PROCESS
REGULATION OF CELL COMMUNICATION
REGULATION OF CELLULAR COMPONENT MOVEMENT
REGULATION OF DEVELOPMENTAL PROCESS
REGULATION OF LOCOMOTION
REGULATION OF MULTICELLULAR ORGANISMAL DEVELOPMENT
REGULATION OF MULTICELLULAR ORGANISMAL PROCESS
REGULATION OF SIGNALING
SYSTEM DEVELOPMENT
TISSUE DEVELOPMENT
TUBE DEVELOPMENT
TUBE MORPHOGENESIS
VASCULATURE DEVELOPMENT
M
EC
A
O
Cell Type
−log10(Adj−p)
40
30
20
10
Genes (n)
50
100
150

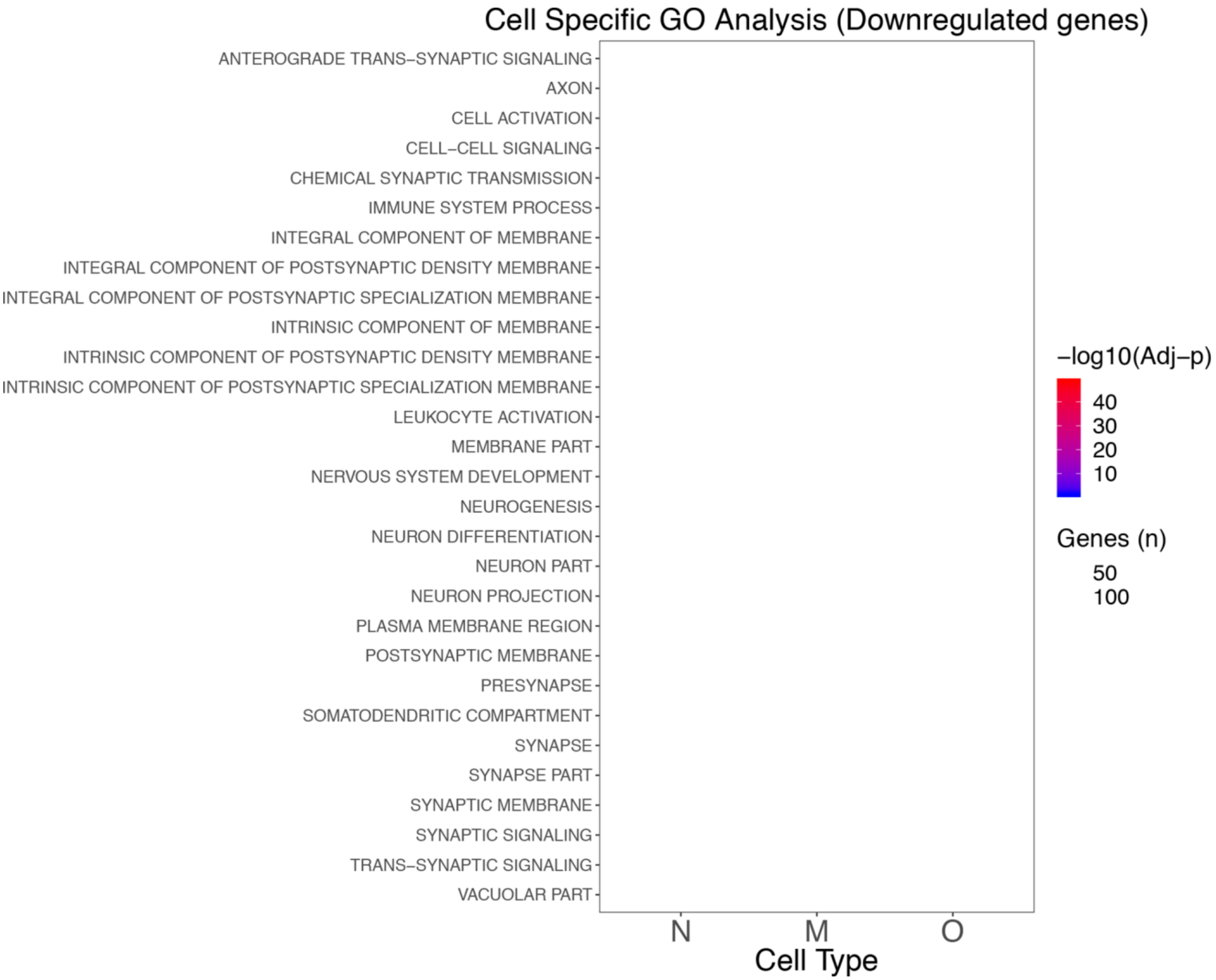

Supplementary Figure 5 (3 pages). Top 15 significant GO classes identified in the different cell types combining the results for the seven brain regions analyzed. The color scale indicates the significance (blue to red as the significance increases), whereas the size indicates the number of genes in that specific enriched class. Page 1: Upregulated and Downregulated genes. Page 2: Upregulated genes. Page 3: Downregulated genes.

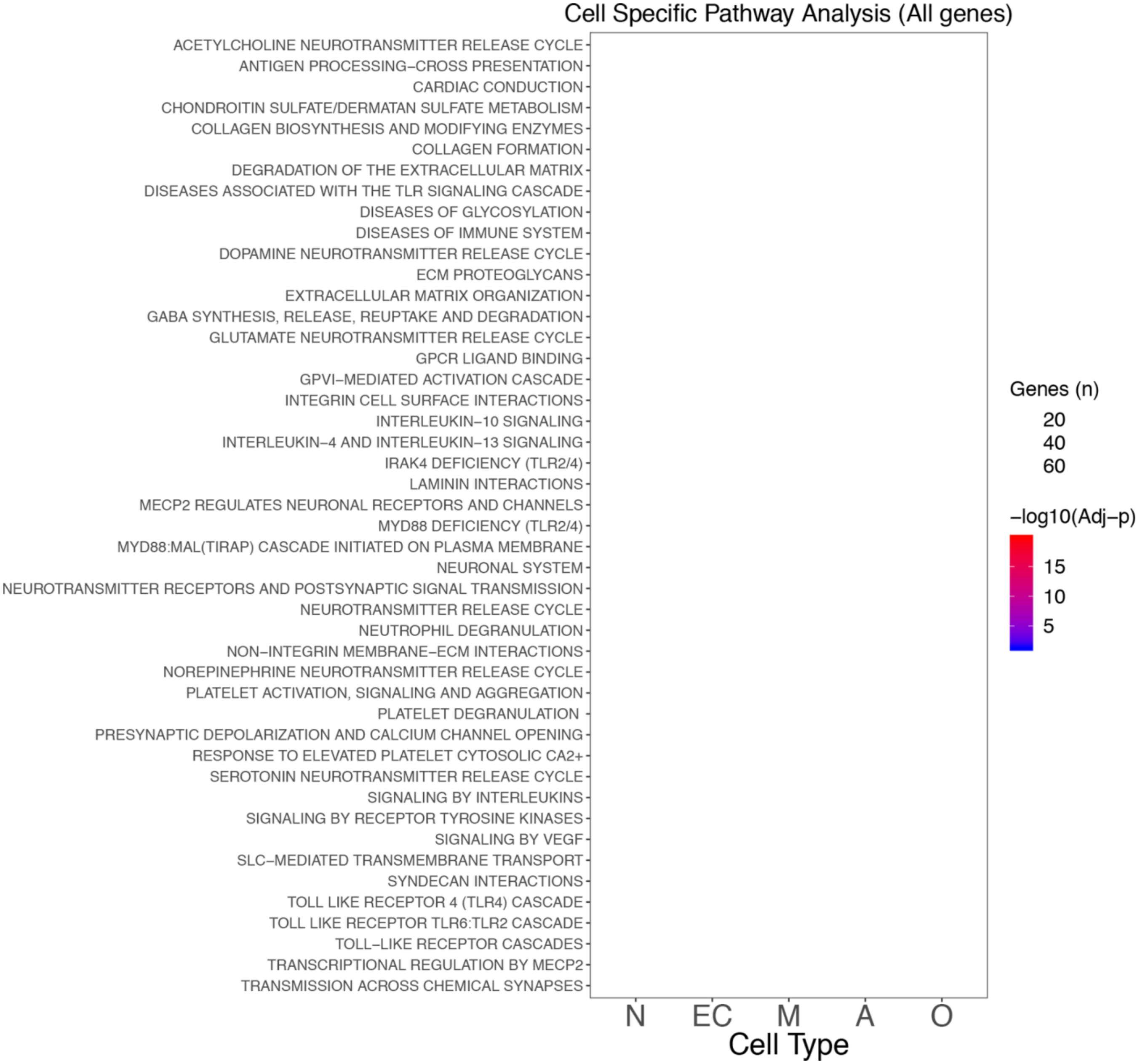
Cell Specific Pathway Analysis (All genes)
ACETYLCHOLINE NEUROTRANSMITTER RELEASE CYCLE
ANTIGEN PROCESSING–CROSS PRESENTATION
CARDIAC CONDUCTION
CHONDROITIN SULFATE/DERMATAN SULFATE METABOLISM
COLLAGEN BIOSYNTHESIS AND MODIFYING ENZYMES
COLLAGEN FORMATION
DEGRADATION OF THE EXTRACELLULAR MATRIX
DISEASES ASSOCIATED WITH THE TLR SIGNALING CASCADE
DISEASES OF GLYCOSYLATION
DISEASES OF IMMUNE SYSTEM
DOPAMINE NEUROTRANSMITTER RELEASE CYCLE
ECM PROTEOGLYCANS
EXTRACELLULAR MATRIX ORGANIZATION
GABA SYNTHESIS, RELEASE, REUPTAKE AND DEGRADATION
GLUTAMATE NEUROTRANSMITTER RELEASE CYCLE
GPCR LIGAND BINDING
GPVI–MEDIATED ACTIVATION CASCADE
INTEGRIN CELL SURFACE INTERACTIONS
INTERLEUKIN–10 SIGNALING
INTERLEUKIN–4 AND INTERLEUKIN–13 SIGNALING
IRAK4 DEFICIENCY (TLR2/4)
LAMININ INTERACTIONS
MECP2 REGULATES NEURONAL RECEPTORS AND CHANNELS
MYD88 DEFICIENCY (TLR2/4)
MYD88:MAL(TIRAP) CASCADE INITIATED ON PLASMA MEMBRANE
NEURONAL SYSTEM
NEUROTRANSMITTER RECEPTORS AND POSTSYNAPTIC SIGNAL TRANSMISSION
NEUROTRANSMITTER RELEASE CYCLE
NEUTROPHIL DEGRANULATION
NON–INTEGRIN MEMBRANE–ECM INTERACTIONS
NOREPINEPHRINE NEUROTRANSMITTER RELEASE CYCLE
PLATELET ACTIVATION, SIGNALING AND AGGREGATION
PLATELET DEGRANULATION
PRESYNAPTIC DEPOLARIZATION AND CALCIUM CHANNEL OPENING
RESPONSE TO ELEVATED PLATELET CYTOSOLIC CA2+
SEROTONIN NEUROTRANSMITTER RELEASE CYCLE
SIGNALING BY INTERLEUKINS
SIGNALING BY RECEPTOR TYROSINE KINASES
SIGNALING BY VEGF
SLC–MEDIATED TRANSMEMBRANE TRANSPORT
SYNDECAN INTERACTIONS
TOLL LIKE RECEPTOR 4 (TLR4) CASCADE
TOLL LIKE RECEPTOR TLR6:TLR2 CASCADE
TOLL–LIKE RECEPTOR CASCADES
TRANSCRIPTIONAL REGULATION BY MECP2
TRANSMISSION ACROSS CHEMICAL SYNAPSES
N
EC
M
A
O
Cell Type
Genes (n)
20
40
60
–log10(Adj–p)
15
10
5

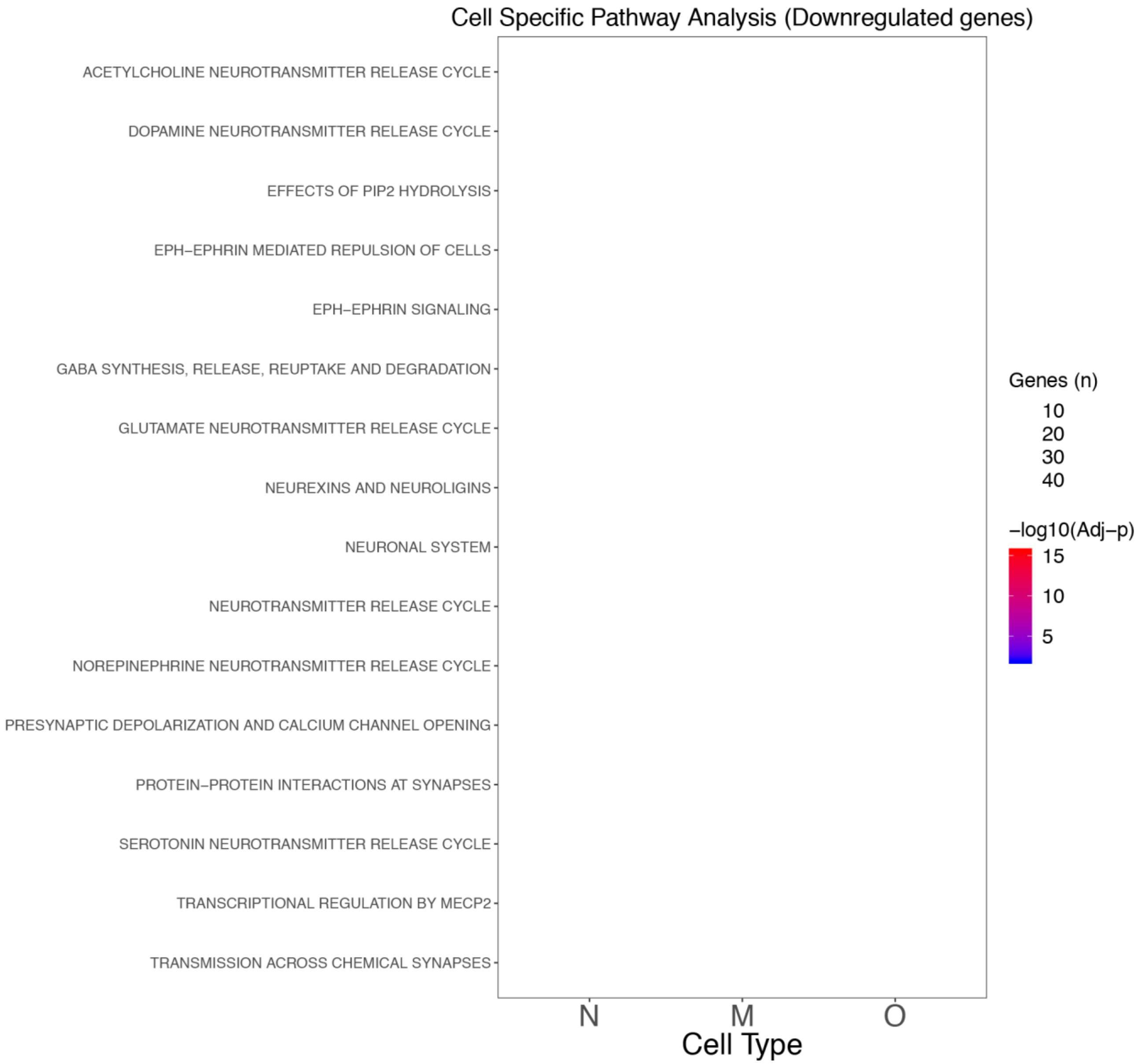

Cell Specific Pathway Analysis (Downregulated genes)
ACETYLCHOLINE NEUROTRANSMITTER RELEASE CYCLE
DOPAMINE NEUROTRANSMITTER RELEASE CYCLE
EFFECTS OF PIP2 HYDROLYSIS
EPH−EPHRIN MEDIATED REPULSION OF CELLS
EPH−EPHRIN SIGNALING
GABA SYNTHESIS, RELEASE, REUPTAKE AND DEGRADATION
GLUTAMATE NEUROTRANSMITTER RELEASE CYCLE
NEUREXINS AND NEUROLIGINS
NEURONAL SYSTEM
NEUROTRANSMITTER RELEASE CYCLE
NOREPINEPHRINE NEUROTRANSMITTER RELEASE CYCLE
PRESYNAPTIC DEPOLARIZATION AND CALCIUM CHANNEL OPENING
PROTEIN−PROTEIN INTERACTIONS AT SYNAPSES
SEROTONIN NEUROTRANSMITTER RELEASE CYCLE
TRANSCRIPTIONAL REGULATION BY MECP2
TRANSMISSION ACROSS CHEMICAL SYNAPSES
N
M
O
Cell Type
Genes (n)
10
20
30
40
−log10(Adj−p)
15
10
5

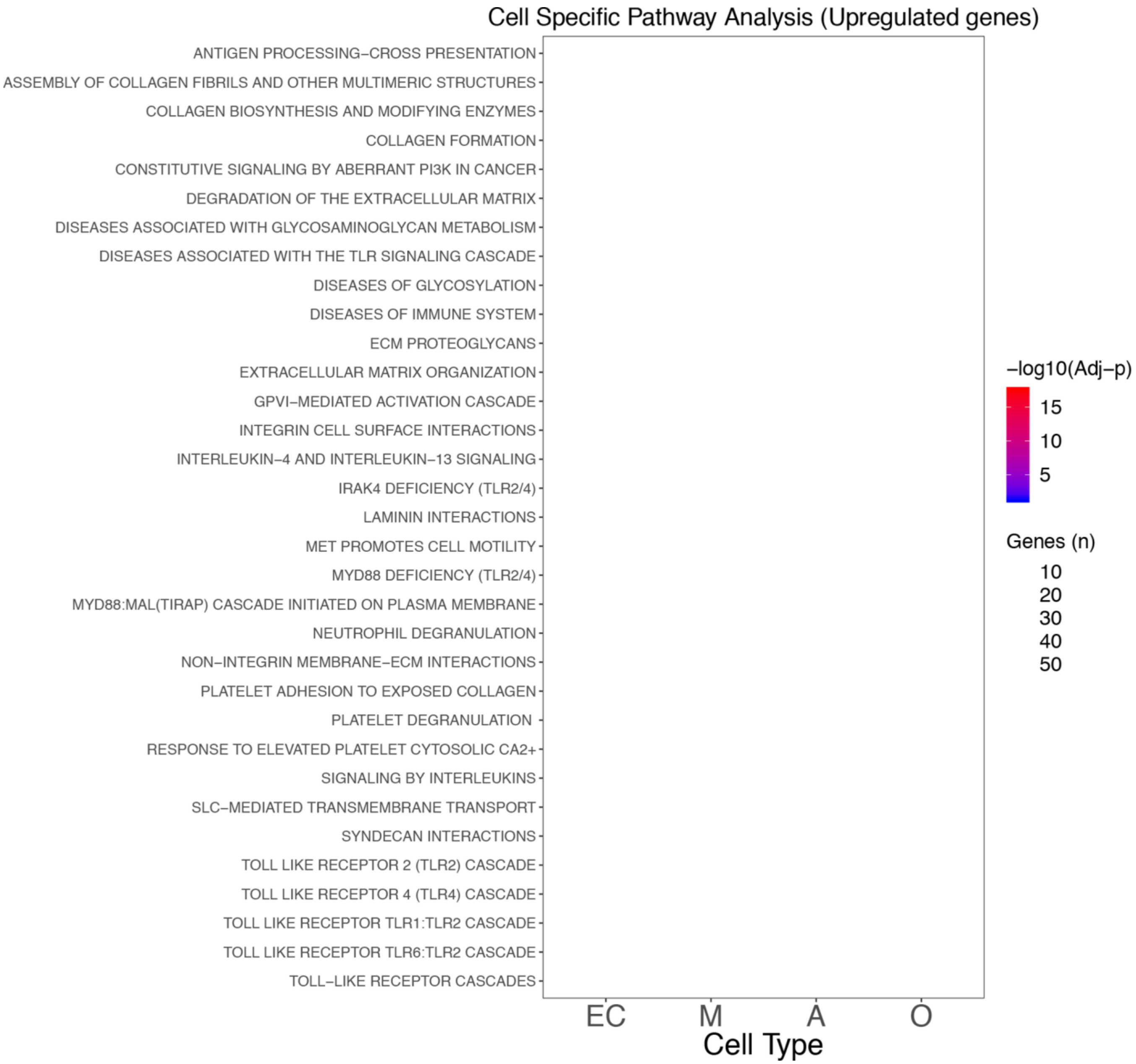

Supplementary Figure 6 (3 pages). Top 15 significant REACTOME pathways identified in the different cell types combining the results for the seven brain regions analyzed. The color scale indicates the significance (blue to red as the significance increases), whereas the size shows the number of genes in that specific enriched class. Page 1: Upregulated and Downregulated genes (same as Figure 4). Page 2: Upregulated genes. Page 3: Downregulated genes.

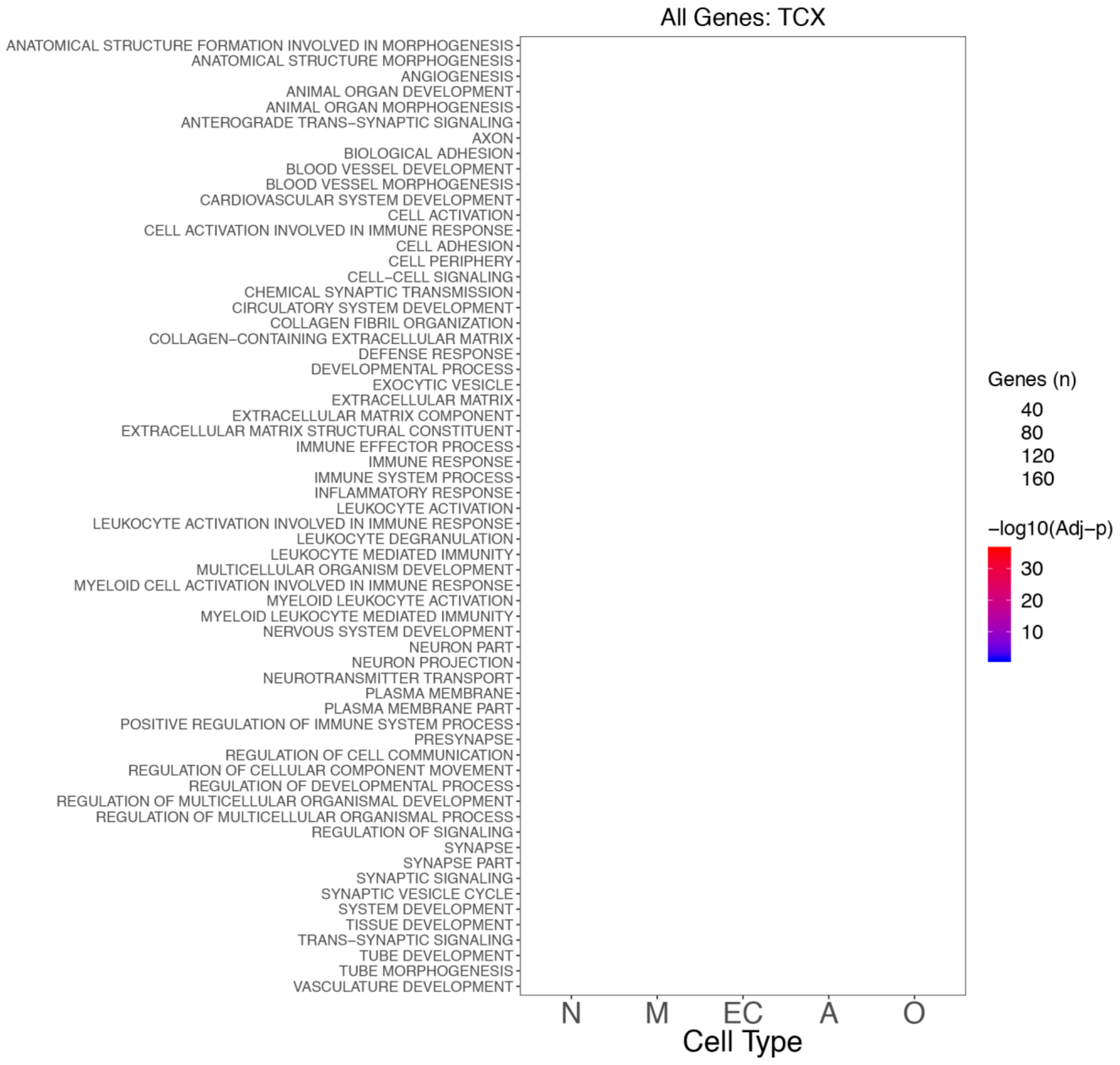

All Genes: TCX
ANATOMICAL STRUCTURE FORMATION INVOLVED IN MORPHOGENESIS
ANATOMICAL STRUCTURE MORPHOGENESIS
ANGIOGENESIS
ANIMAL ORGAN DEVELOPMENT
ANIMAL ORGAN MORPHOGENESIS
ANTEROGRADE TRANS–SYNAPTIC SIGNALING
AXON
BIOLOGICAL ADHESION
BLOOD VESSEL DEVELOPMENT
BLOOD VESSEL MORPHOGENESIS
CARDIOVASCULAR SYSTEM DEVELOPMENT
CELL ACTIVATION
CELL ACTIVATION INVOLVED IN IMMUNE RESPONSE
CELL ADHESION
CELL PERIPHERY
CELL–CELL SIGNALING
CHEMICAL SYNAPTIC TRANSMISSION
CIRCULATORY SYSTEM DEVELOPMENT
COLLAGEN FIBRIL ORGANIZATION
COLLAGEN–CONTAINING EXTRACELLULAR MATRIX
DEFENSE RESPONSE
DEVELOPMENTAL PROCESS
EXOCYTIC VESICLE
EXTRACELLULAR MATRIX
EXTRACELLULAR MATRIX COMPONENT
EXTRACELLULAR MATRIX STRUCTURAL CONSTITUENT
IMMUNE EFFECTOR PROCESS
IMMUNE RESPONSE
IMMUNE SYSTEM PROCESS
INFLAMMATORY RESPONSE
LEUKOCYTE ACTIVATION
LEUKOCYTE ACTIVATION INVOLVED IN IMMUNE RESPONSE
LEUKOCYTE DEGRANULATION
LEUKOCYTE MEDIATED IMMUNITY
MULTICELLULAR ORGANISM DEVELOPMENT
MYELOID CELL ACTIVATION INVOLVED IN IMMUNE RESPONSE
MYELOID LEUKOCYTE ACTIVATION
MYELOID LEUKOCYTE MEDIATED IMMUNITY
NERVOUS SYSTEM DEVELOPMENT
NEURON PART
NEURON PROJECTION
NEUROTRANSMITTER TRANSPORT
PLASMA MEMBRANE
PLASMA MEMBRANE PART
POSITIVE REGULATION OF IMMUNE SYSTEM PROCESS
PRESYNAPSE
REGULATION OF CELL COMMUNICATION
REGULATION OF CELLULAR COMPONENT MOVEMENT
REGULATION OF DEVELOPMENTAL PROCESS
REGULATION OF MULTICELLULAR ORGANISMAL DEVELOPMENT
REGULATION OF MULTICELLULAR ORGANISMAL PROCESS
REGULATION OF SIGNALING
SYNAPSE
SYNAPSE PART
SYNAPTIC SIGNALING
SYNAPTIC VESICLE CYCLE
SYSTEM DEVELOPMENT
TISSUE DEVELOPMENT
TRANS–SYNAPTIC SIGNALING
TUBE DEVELOPMENT
TUBE MORPHOGENESIS
VASCULATURE DEVELOPMENT
N
M
EC
A
O
Cell Type
Genes (n)
40
80
120
160
–log10(Adj–p)
30
20
10

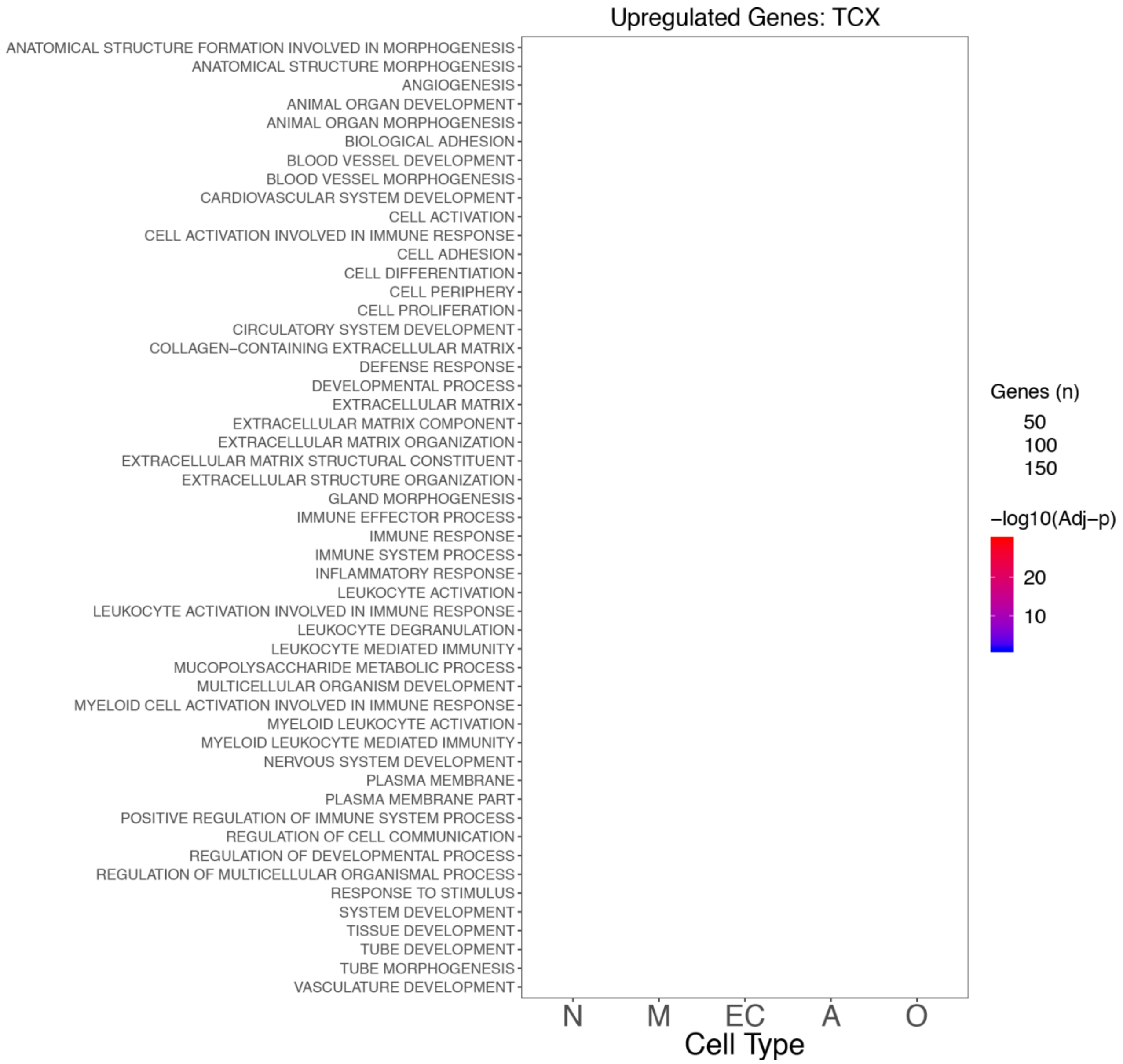
Upregulated Genes: TCX
ANATOMICAL STRUCTURE FORMATION INVOLVED IN MORPHOGENESIS
ANATOMICAL STRUCTURE MORPHOGENESIS
ANGIOGENESIS
ANIMAL ORGAN DEVELOPMENT
ANIMAL ORGAN MORPHOGENESIS
BIOLOGICAL ADHESION
BLOOD VESSEL DEVELOPMENT
BLOOD VESSEL MORPHOGENESIS
CARDIOVASCULAR SYSTEM DEVELOPMENT
CELL ACTIVATION
CELL ACTIVATION INVOLVED IN IMMUNE RESPONSE
CELL ADHESION
CELL DIFFERENTIATION
CELL PERIPHERY
CELL PROLIFERATION
CIRCULATORY SYSTEM DEVELOPMENT
COLLAGEN−CONTAINING EXTRACELLULAR MATRIX
DEFENSE RESPONSE
DEVELOPMENTAL PROCESS
EXTRACELLULAR MATRIX
EXTRACELLULAR MATRIX COMPONENT
EXTRACELLULAR MATRIX ORGANIZATION
EXTRACELLULAR MATRIX STRUCTURAL CONSTITUENT
EXTRACELLULAR STRUCTURE ORGANIZATION
GLAND MORPHOGENESIS
IMMUNE EFFECTOR PROCESS
IMMUNE RESPONSE
IMMUNE SYSTEM PROCESS
INFLAMMATORY RESPONSE
LEUKOCYTE ACTIVATION
LEUKOCYTE ACTIVATION INVOLVED IN IMMUNE RESPONSE
LEUKOCYTE DEGRANULATION
LEUKOCYTE MEDIATED IMMUNITY
MUCOPOLYSACCHARIDE METABOLIC PROCESS
MULTICELLULAR ORGANISM DEVELOPMENT
MYELOID CELL ACTIVATION INVOLVED IN IMMUNE RESPONSE
MYELOID LEUKOCYTE ACTIVATION
MYELOID LEUKOCYTE MEDIATED IMMUNITY
NERVOUS SYSTEM DEVELOPMENT
PLASMA MEMBRANE
PLASMA MEMBRANE PART
POSITIVE REGULATION OF IMMUNE SYSTEM PROCESS
REGULATION OF CELL COMMUNICATION
REGULATION OF DEVELOPMENTAL PROCESS
REGULATION OF MULTICELLULAR ORGANISMAL PROCESS
RESPONSE TO STIMULUS
SYSTEM DEVELOPMENT
TISSUE DEVELOPMENT
TUBE DEVELOPMENT
TUBE MORPHOGENESIS
VASCULATURE DEVELOPMENT
N
M
EC
A
O
Cell Type
Genes (n)
50
100
150
−log10(Adj−p)
20
10

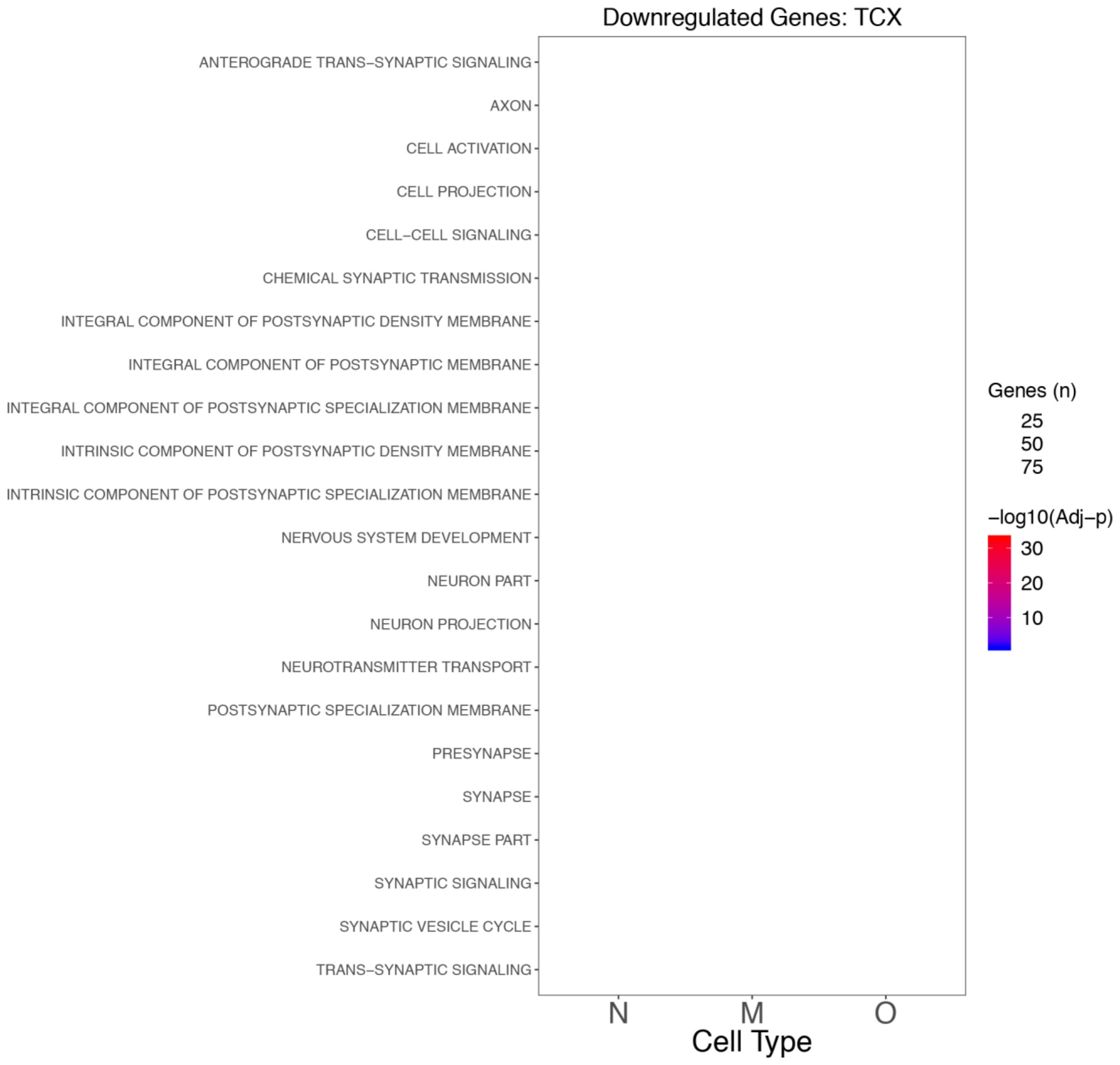
Downregulated Genes: TCX
ANTEROGRADE TRANS–SYNAPTIC SIGNALING
AXON
CELL ACTIVATION
CELL PROJECTION
CELL–CELL SIGNALING
CHEMICAL SYNAPTIC TRANSMISSION
INTEGRAL COMPONENT OF POSTSYNAPTIC DENSITY MEMBRANE
INTEGRAL COMPONENT OF POSTSYNAPTIC MEMBRANE
INTEGRAL COMPONENT OF POSTSYNAPTIC SPECIALIZATION MEMBRANE
INTRINSIC COMPONENT OF POSTSYNAPTIC DENSITY MEMBRANE
INTRINSIC COMPONENT OF POSTSYNAPTIC SPECIALIZATION MEMBRANE
NERVOUS SYSTEM DEVELOPMENT
NEURON PART
NEURON PROJECTION
NEUROTRANSMITTER TRANSPORT
POSTSYNAPTIC SPECIALIZATION MEMBRANE
PRESYNAPSE
SYNAPSE
SYNAPSE PART
SYNAPTIC SIGNALING
SYNAPTIC VESICLE CYCLE
TRANS–SYNAPTIC SIGNALING
N
M
O
Cell Type
Genes (n)
25
50
75
–log10(Adj–p)
30
20
10

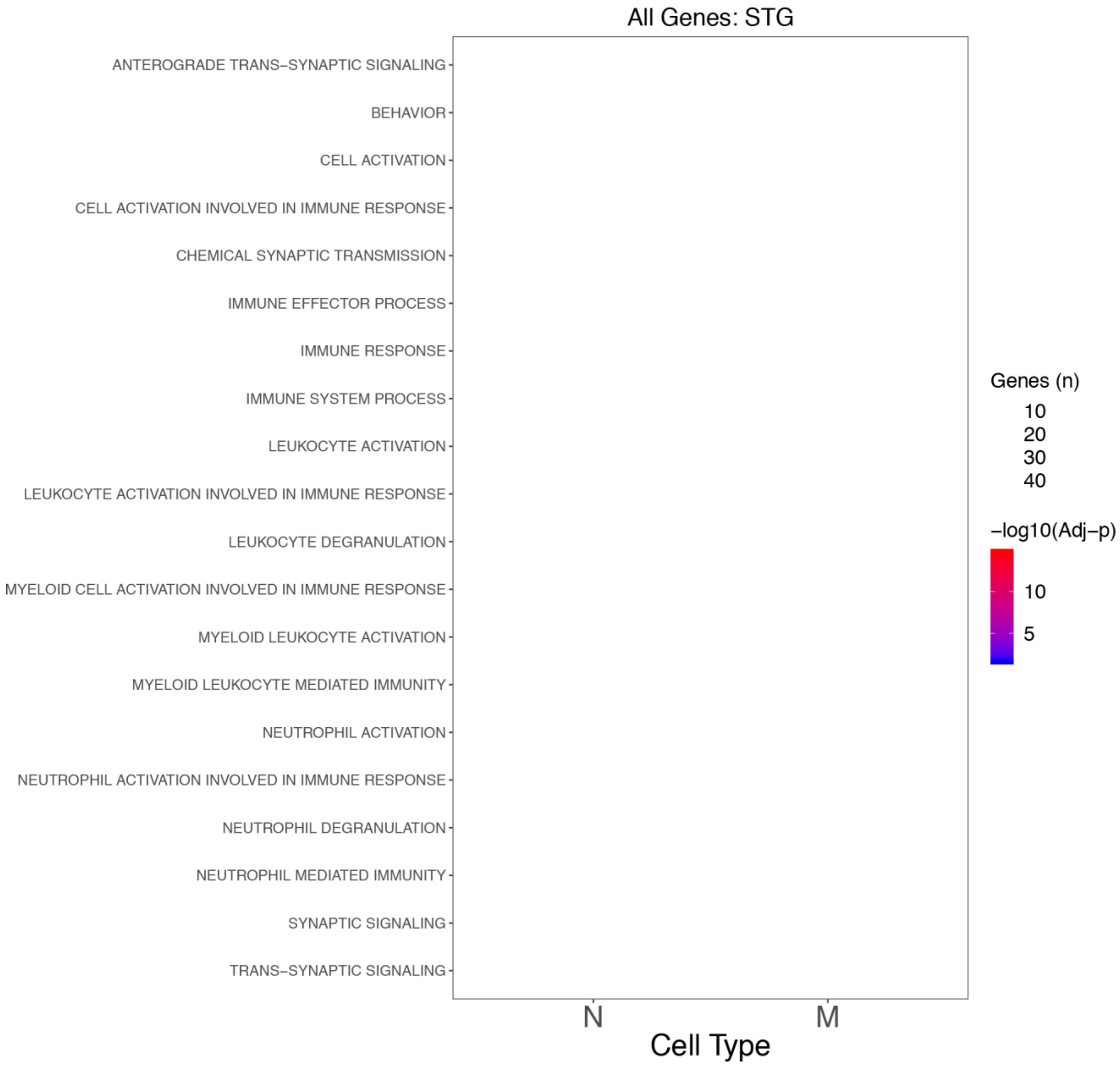
All Genes: STG
ANTEROGRADE TRANS–SYNAPTIC SIGNALING
BEHAVIOR
CELL ACTIVATION
CELL ACTIVATION INVOLVED IN IMMUNE RESPONSE
CHEMICAL SYNAPTIC TRANSMISSION
IMMUNE EFFECTOR PROCESS
IMMUNE RESPONSE
IMMUNE SYSTEM PROCESS
LEUKOCYTE ACTIVATION
LEUKOCYTE ACTIVATION INVOLVED IN IMMUNE RESPONSE
LEUKOCYTE DEGRANULATION
MYELOID CELL ACTIVATION INVOLVED IN IMMUNE RESPONSE
MYELOID LEUKOCYTE ACTIVATION
MYELOID LEUKOCYTE MEDIATED IMMUNITY
NEUTROPHIL ACTIVATION
NEUTROPHIL ACTIVATION INVOLVED IN IMMUNE RESPONSE
NEUTROPHIL DEGRANULATION
NEUTROPHIL MEDIATED IMMUNITY
SYNAPTIC SIGNALING
TRANS–SYNAPTIC SIGNALING
N
M
Cell Type
Genes (n)
10
20
30
40
–log10(Adj–p)
10
5

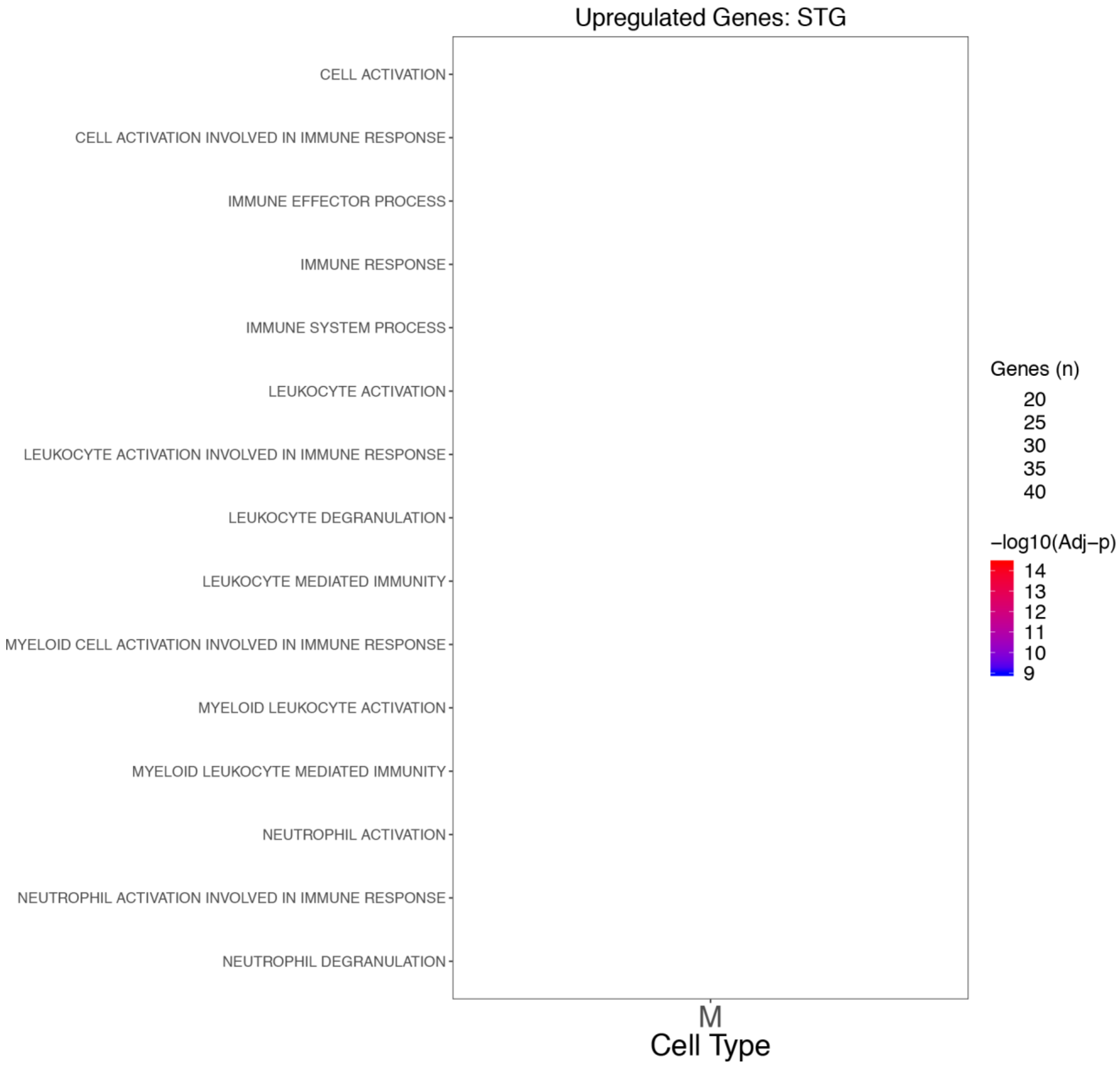

Upregulated Genes: STG
CELL ACTIVATION
CELL ACTIVATION INVOLVED IN IMMUNE RESPONSE
IMMUNE EFFECTOR PROCESS
IMMUNE RESPONSE
IMMUNE SYSTEM PROCESS
LEUKOCYTE ACTIVATION
LEUKOCYTE ACTIVATION INVOLVED IN IMMUNE RESPONSE
LEUKOCYTE DEGRANULATION
LEUKOCYTE MEDIATED IMMUNITY
MYELOID CELL ACTIVATION INVOLVED IN IMMUNE RESPONSE
MYELOID LEUKOCYTE ACTIVATION
MYELOID LEUKOCYTE MEDIATED IMMUNITY
NEUTROPHIL ACTIVATION
NEUTROPHIL ACTIVATION INVOLVED IN IMMUNE RESPONSE
NEUTROPHIL DEGRANULATION
M
Cell Type
Genes (n)
20
25
30
35
40
−log10(Adj−p)
14
13
12
11
10
9

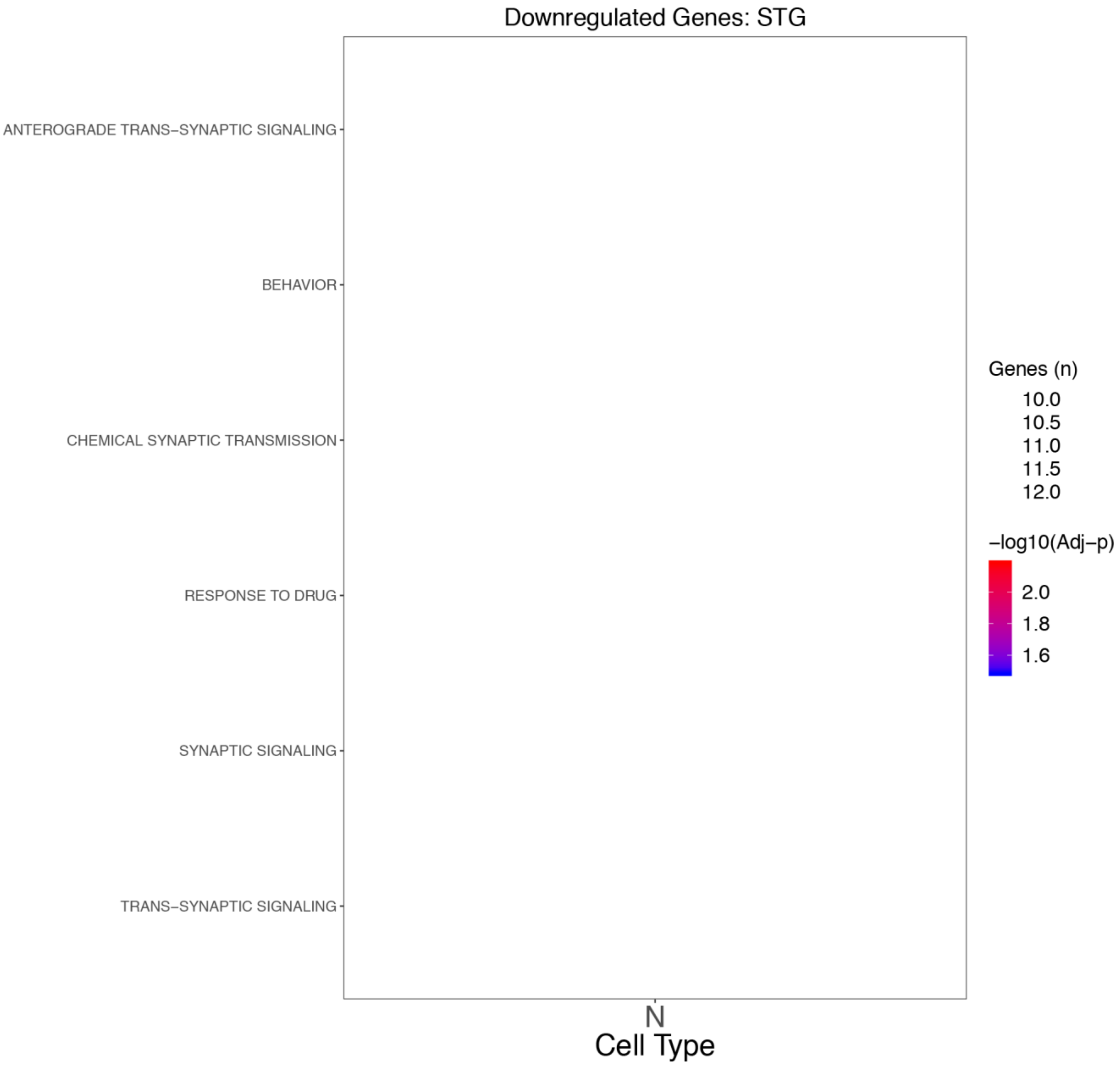
Downregulated Genes: STG
ANTEROGRADE TRANS−SYNAPTIC SIGNALING
BEHAVIOR
CHEMICAL SYNAPTIC TRANSMISSION
RESPONSE TO DRUG
SYNAPTIC SIGNALING
TRANS−SYNAPTIC SIGNALING
N
Cell Type
Genes (n)
10.0
10.5
11.0
11.5
12.0
−log10(Adj−p)
2.0
1.8
1.6

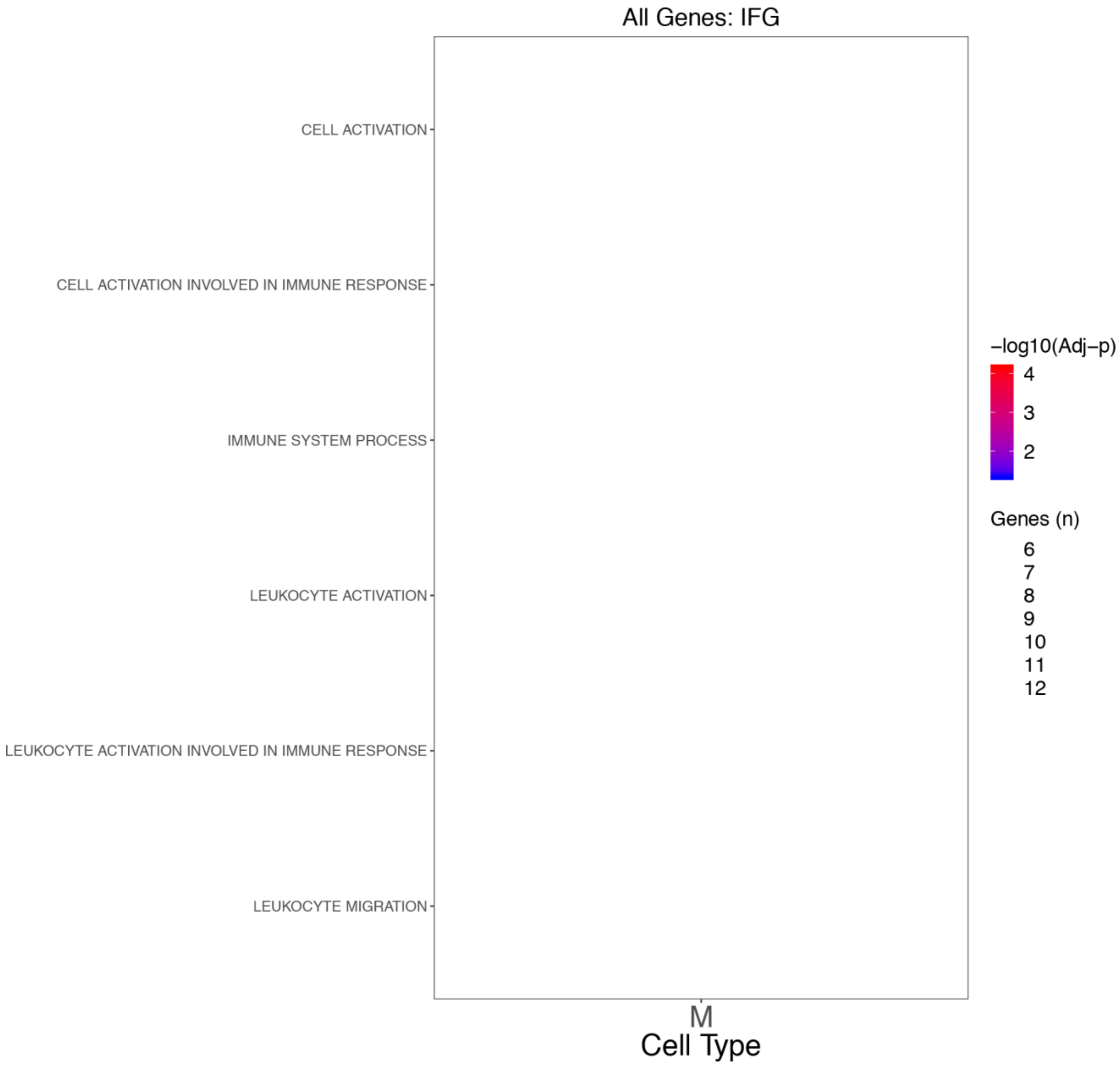
All Genes: IFG
CELL ACTIVATION
CELL ACTIVATION INVOLVED IN IMMUNE RESPONSE
IMMUNE SYSTEM PROCESS
LEUKOCYTE ACTIVATION
LEUKOCYTE ACTIVATION INVOLVED IN IMMUNE RESPONSE
LEUKOCYTE MIGRATION
M
Cell Type
−log10(Adj−p)
4
3
2
Genes (n)
6
7
8
9
10
11
12

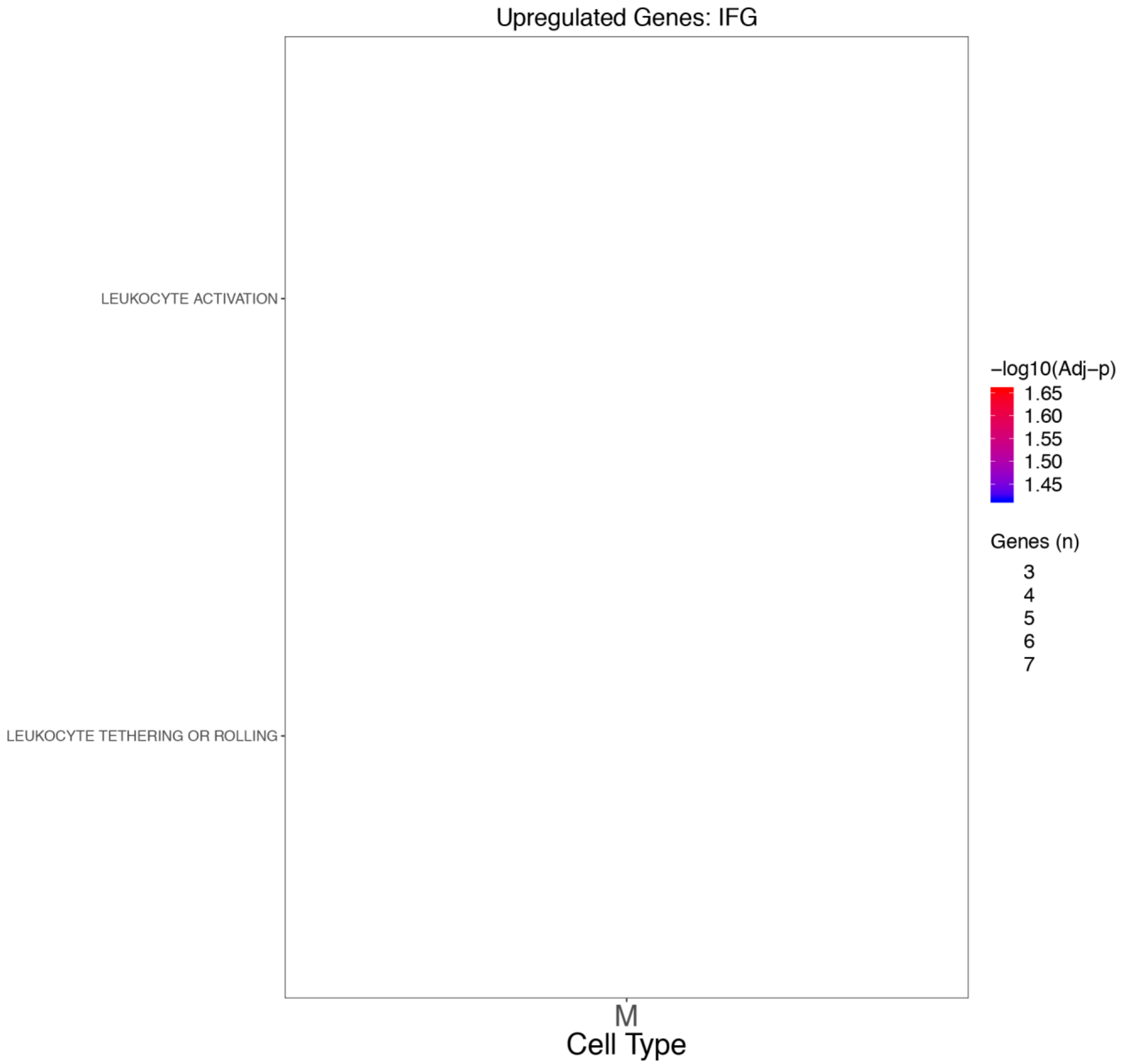
Upregulated Genes: IFG
LEUKOCYTE ACTIVATION
LEUKOCYTE TETHERING OR ROLLING
−log10(Adj−p)
1.65
1.60
1.55
1.50
1.45
Genes (n)
3
4
5
6
7
M
Cell Type

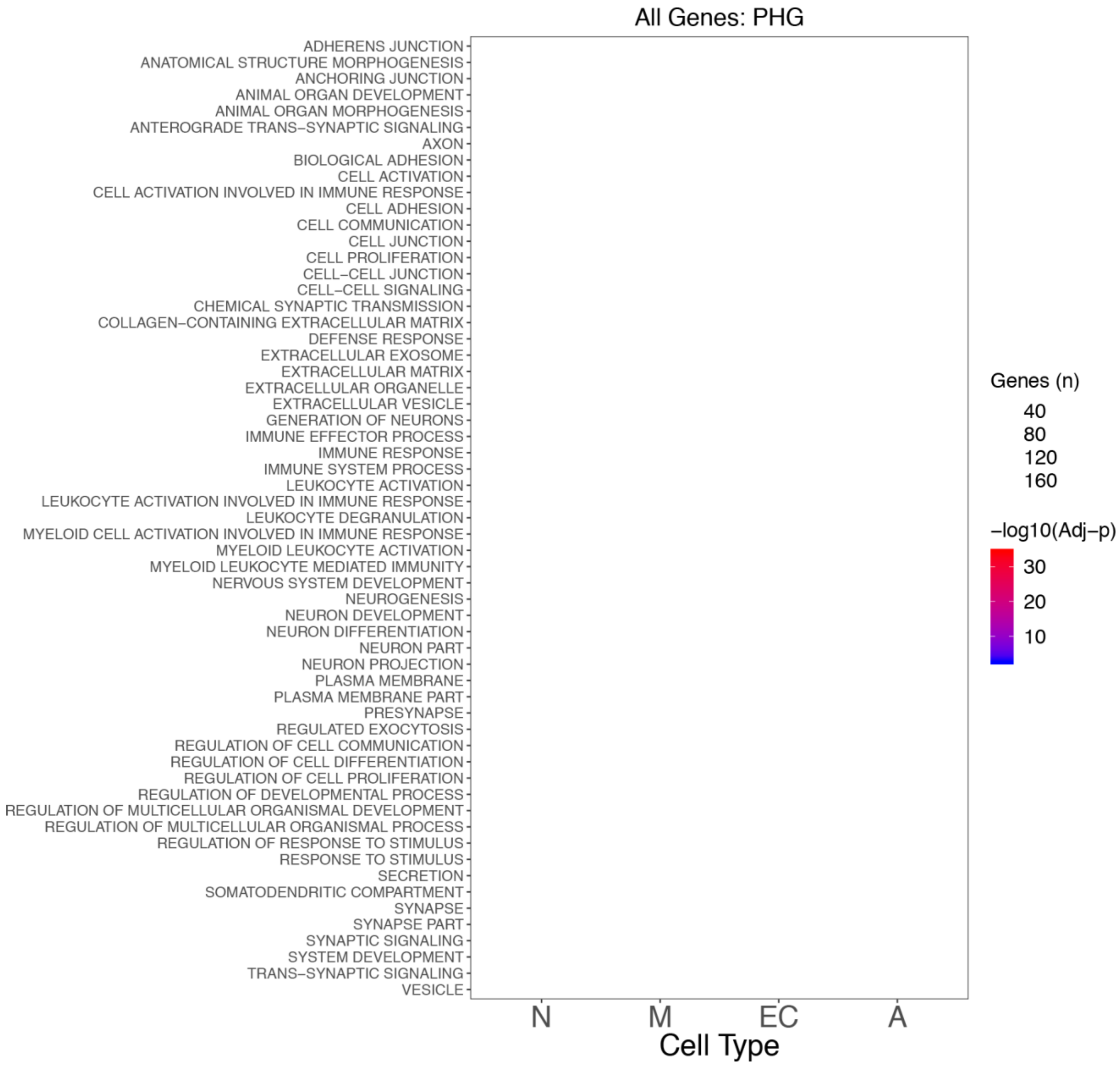

All Genes: PHG
ADHERENS JUNCTION
ANATOMICAL STRUCTURE MORPHOGENESIS
ANCHORING JUNCTION
ANIMAL ORGAN DEVELOPMENT
ANIMAL ORGAN MORPHOGENESIS
ANTEROGRADE TRANS–SYNAPTIC SIGNALING
AXON
BIOLOGICAL ADHESION
CELL ACTIVATION
CELL ACTIVATION INVOLVED IN IMMUNE RESPONSE
CELL ADHESION
CELL COMMUNICATION
CELL JUNCTION
CELL PROLIFERATION
CELL–CELL JUNCTION
CELL–CELL SIGNALING
CHEMICAL SYNAPTIC TRANSMISSION
COLLAGEN–CONTAINING EXTRACELLULAR MATRIX
DEFENSE RESPONSE
EXTRACELLULAR EXOSOME
EXTRACELLULAR MATRIX
EXTRACELLULAR ORGANELLE
EXTRACELLULAR VESICLE
GENERATION OF NEURONS
IMMUNE EFFECTOR PROCESS
IMMUNE RESPONSE
IMMUNE SYSTEM PROCESS
LEUKOCYTE ACTIVATION
LEUKOCYTE ACTIVATION INVOLVED IN IMMUNE RESPONSE
LEUKOCYTE DEGRANULATION
MYELOID CELL ACTIVATION INVOLVED IN IMMUNE RESPONSE
MYELOID LEUKOCYTE ACTIVATION
MYELOID LEUKOCYTE MEDIATED IMMUNITY
NERVOUS SYSTEM DEVELOPMENT
NEUROGENESIS
NEURON DEVELOPMENT
NEURON DIFFERENTIATION
NEURON PART
NEURON PROJECTION
PLASMA MEMBRANE
PLASMA MEMBRANE PART
PRESYNAPSE
REGULATED EXOCYTOSIS
REGULATION OF CELL COMMUNICATION
REGULATION OF CELL DIFFERENTIATION
REGULATION OF CELL PROLIFERATION
REGULATION OF DEVELOPMENTAL PROCESS
REGULATION OF MULTICELLULAR ORGANISMAL DEVELOPMENT
REGULATION OF MULTICELLULAR ORGANISMAL PROCESS
REGULATION OF RESPONSE TO STIMULUS
RESPONSE TO STIMULUS
SECRETION
SOMATODENDRITIC COMPARTMENT
SYNAPSE
SYNAPSE PART
SYNAPTIC SIGNALING
SYSTEM DEVELOPMENT
TRANS–SYNAPTIC SIGNALING
VESICLE
N
M
EC
A
Cell Type
Genes (n)
40
80
120
160
–log10(Adj–p)
30
20
10

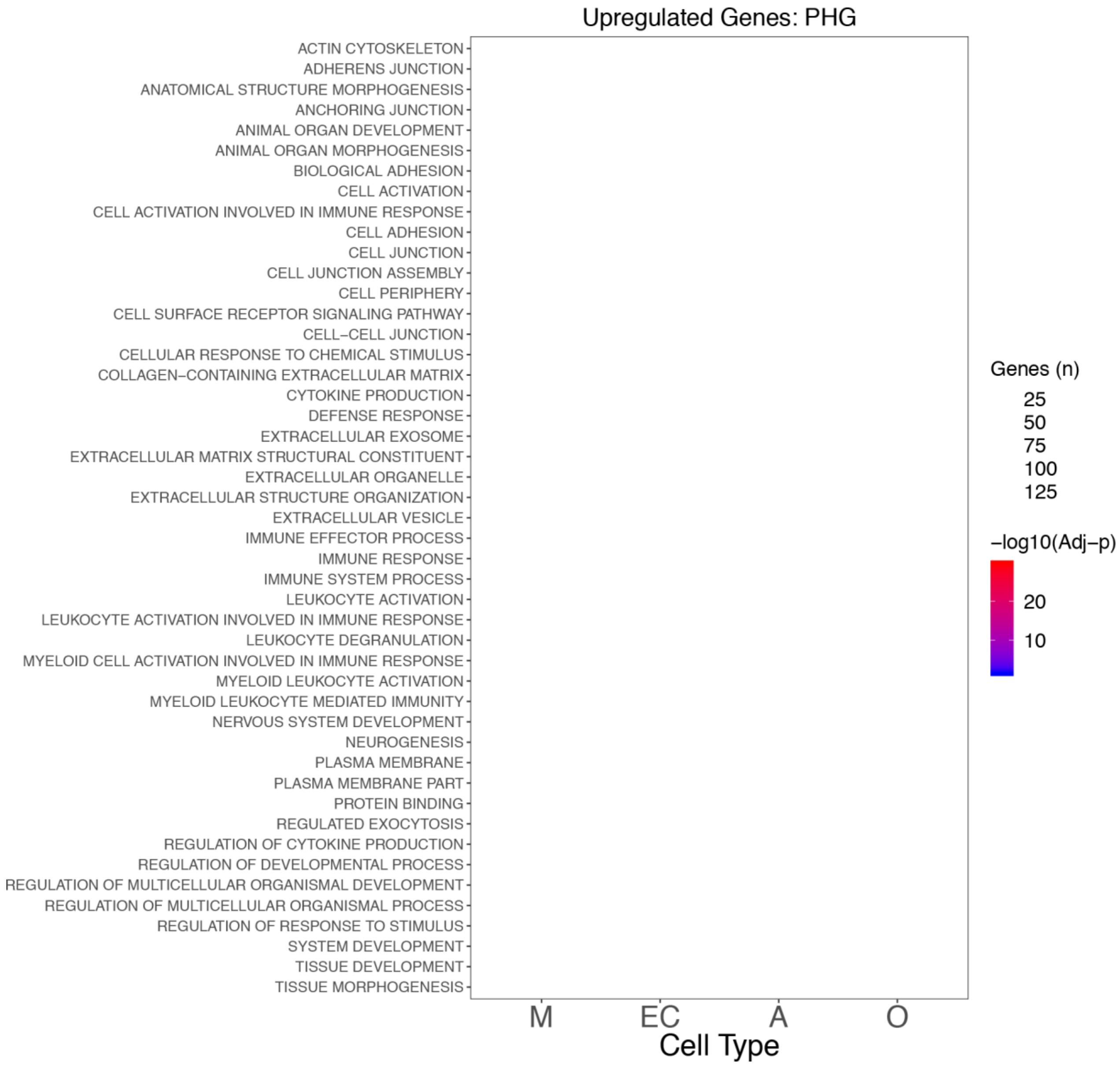
Upregulated Genes: PHG
ACTIN CYTOSKELETON
ADHERENS JUNCTION
ANATOMICAL STRUCTURE MORPHOGENESIS
ANCHORING JUNCTION
ANIMAL ORGAN DEVELOPMENT
ANIMAL ORGAN MORPHOGENESIS
BIOLOGICAL ADHESION
CELL ACTIVATION
CELL ACTIVATION INVOLVED IN IMMUNE RESPONSE
CELL ADHESION
CELL JUNCTION
CELL JUNCTION ASSEMBLY
CELL PERIPHERY
CELL SURFACE RECEPTOR SIGNALING PATHWAY
CELL–CELL JUNCTION
CELLULAR RESPONSE TO CHEMICAL STIMULUS
COLLAGEN–CONTAINING EXTRACELLULAR MATRIX
CYTOKINE PRODUCTION
DEFENSE RESPONSE
EXTRACELLULAR EXOSOME
EXTRACELLULAR MATRIX STRUCTURAL CONSTITUENT
EXTRACELLULAR ORGANELLE
EXTRACELLULAR STRUCTURE ORGANIZATION
EXTRACELLULAR VESICLE
IMMUNE EFFECTOR PROCESS
IMMUNE RESPONSE
IMMUNE SYSTEM PROCESS
LEUKOCYTE ACTIVATION
LEUKOCYTE ACTIVATION INVOLVED IN IMMUNE RESPONSE
LEUKOCYTE DEGRANULATION
MYELOID CELL ACTIVATION INVOLVED IN IMMUNE RESPONSE
MYELOID LEUKOCYTE ACTIVATION
MYELOID LEUKOCYTE MEDIATED IMMUNITY
NERVOUS SYSTEM DEVELOPMENT
NEUROGENESIS
PLASMA MEMBRANE
PLASMA MEMBRANE PART
PROTEIN BINDING
REGULATED EXOCYTOSIS
REGULATION OF CYTOKINE PRODUCTION
REGULATION OF DEVELOPMENTAL PROCESS
REGULATION OF MULTICELLULAR ORGANISMAL DEVELOPMENT
REGULATION OF MULTICELLULAR ORGANISMAL PROCESS
REGULATION OF RESPONSE TO STIMULUS
SYSTEM DEVELOPMENT
TISSUE DEVELOPMENT
TISSUE MORPHOGENESIS
M
EC
A
O
Cell Type
Genes (n)
25
50
75
100
125
–log10(Adj–p)
20
10

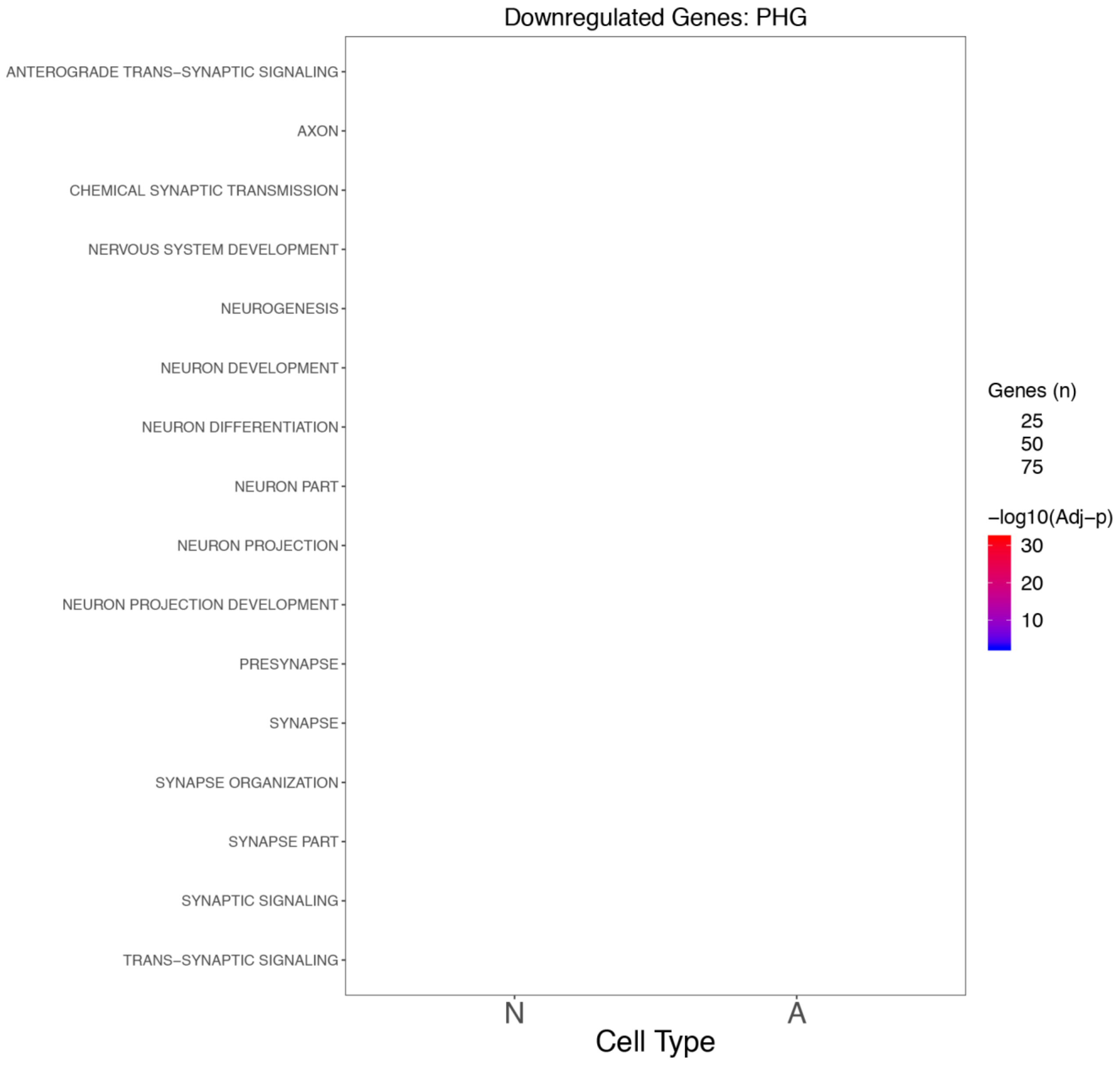
Downregulated Genes: PHG
ANTEROGRADE TRANS−SYNAPTIC SIGNALING
AXON
CHEMICAL SYNAPTIC TRANSMISSION
NERVOUS SYSTEM DEVELOPMENT
NEUROGENESIS
NEURON DEVELOPMENT
NEURON DIFFERENTIATION
NEURON PART
NEURON PROJECTION
NEURON PROJECTION DEVELOPMENT
PRESYNAPSE
SYNAPSE
SYNAPSE ORGANIZATION
SYNAPSE PART
SYNAPTIC SIGNALING
TRANS−SYNAPTIC SIGNALING
N
A
Cell Type
Genes (n)
25
50
75
−log10(Adj−p)
30
20
10

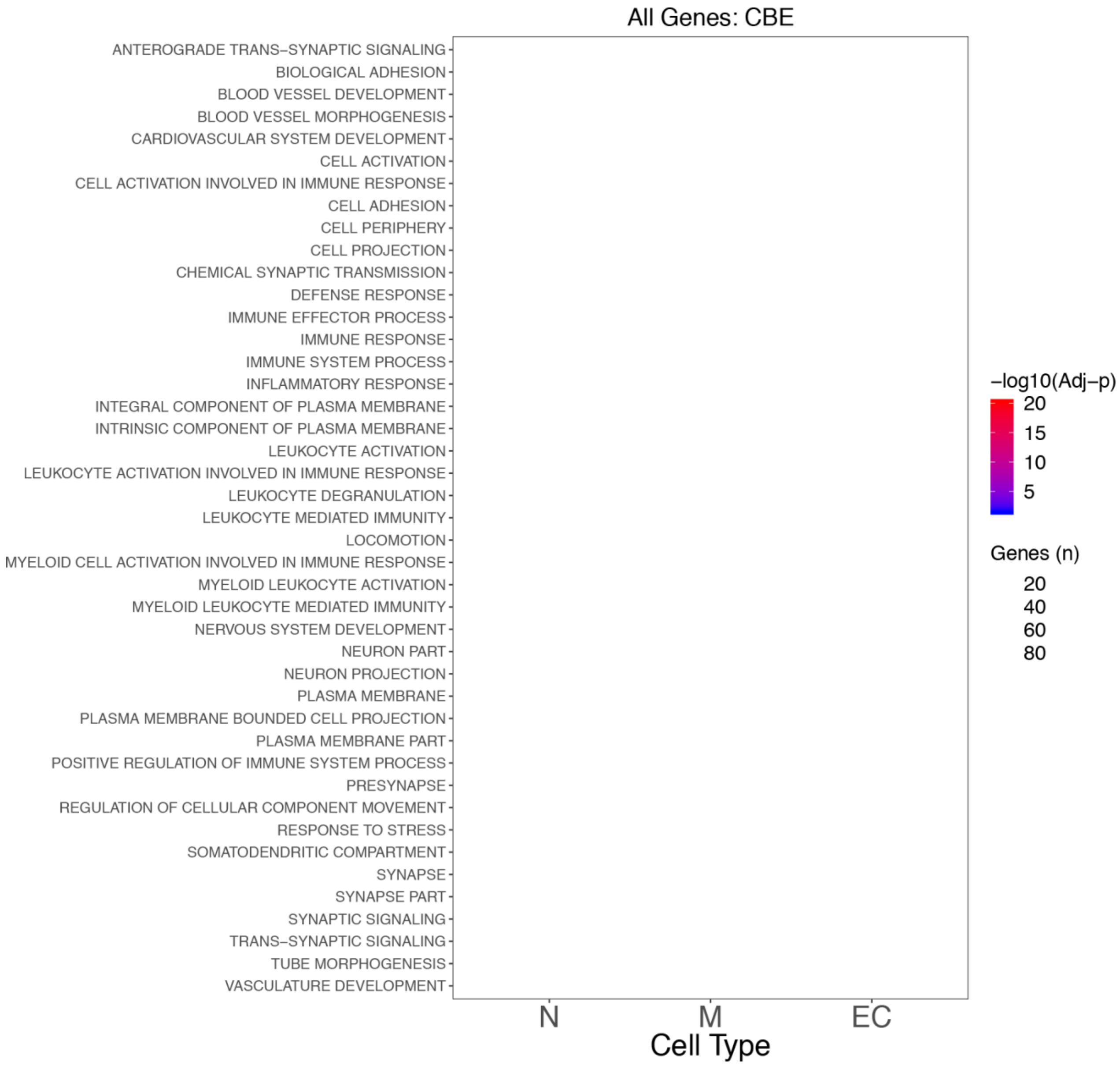

All Genes: CBE
ANTEROGRADE TRANS−SYNAPTIC SIGNALING
BIOLOGICAL ADHESION
BLOOD VESSEL DEVELOPMENT
BLOOD VESSEL MORPHOGENESIS
CARDIOVASCULAR SYSTEM DEVELOPMENT
CELL ACTIVATION
CELL ACTIVATION INVOLVED IN IMMUNE RESPONSE
CELL ADHESION
CELL PERIPHERY
CELL PROJECTION
CHEMICAL SYNAPTIC TRANSMISSION
DEFENSE RESPONSE
IMMUNE EFFECTOR PROCESS
IMMUNE RESPONSE
IMMUNE SYSTEM PROCESS
INFLAMMATORY RESPONSE
INTEGRAL COMPONENT OF PLASMA MEMBRANE
INTRINSIC COMPONENT OF PLASMA MEMBRANE
LEUKOCYTE ACTIVATION
LEUKOCYTE ACTIVATION INVOLVED IN IMMUNE RESPONSE
LEUKOCYTE DEGRANULATION
LEUKOCYTE MEDIATED IMMUNITY
LOCOMOTION
MYELOID CELL ACTIVATION INVOLVED IN IMMUNE RESPONSE
MYELOID LEUKOCYTE ACTIVATION
MYELOID LEUKOCYTE MEDIATED IMMUNITY
NERVOUS SYSTEM DEVELOPMENT
NEURON PART
NEURON PROJECTION
PLASMA MEMBRANE
PLASMA MEMBRANE BOUNDED CELL PROJECTION
PLASMA MEMBRANE PART
POSITIVE REGULATION OF IMMUNE SYSTEM PROCESS
PRESYNAPSE
REGULATION OF CELLULAR COMPONENT MOVEMENT
RESPONSE TO STRESS
SOMATODENDRITIC COMPARTMENT
SYNAPSE
SYNAPSE PART
SYNAPTIC SIGNALING
TRANS−SYNAPTIC SIGNALING
TUBE MORPHOGENESIS
VASCULATURE DEVELOPMENT
N
M
EC
Cell Type
−log10(Adj−p)
20
15
10
5
Genes (n)
20
40
60
80

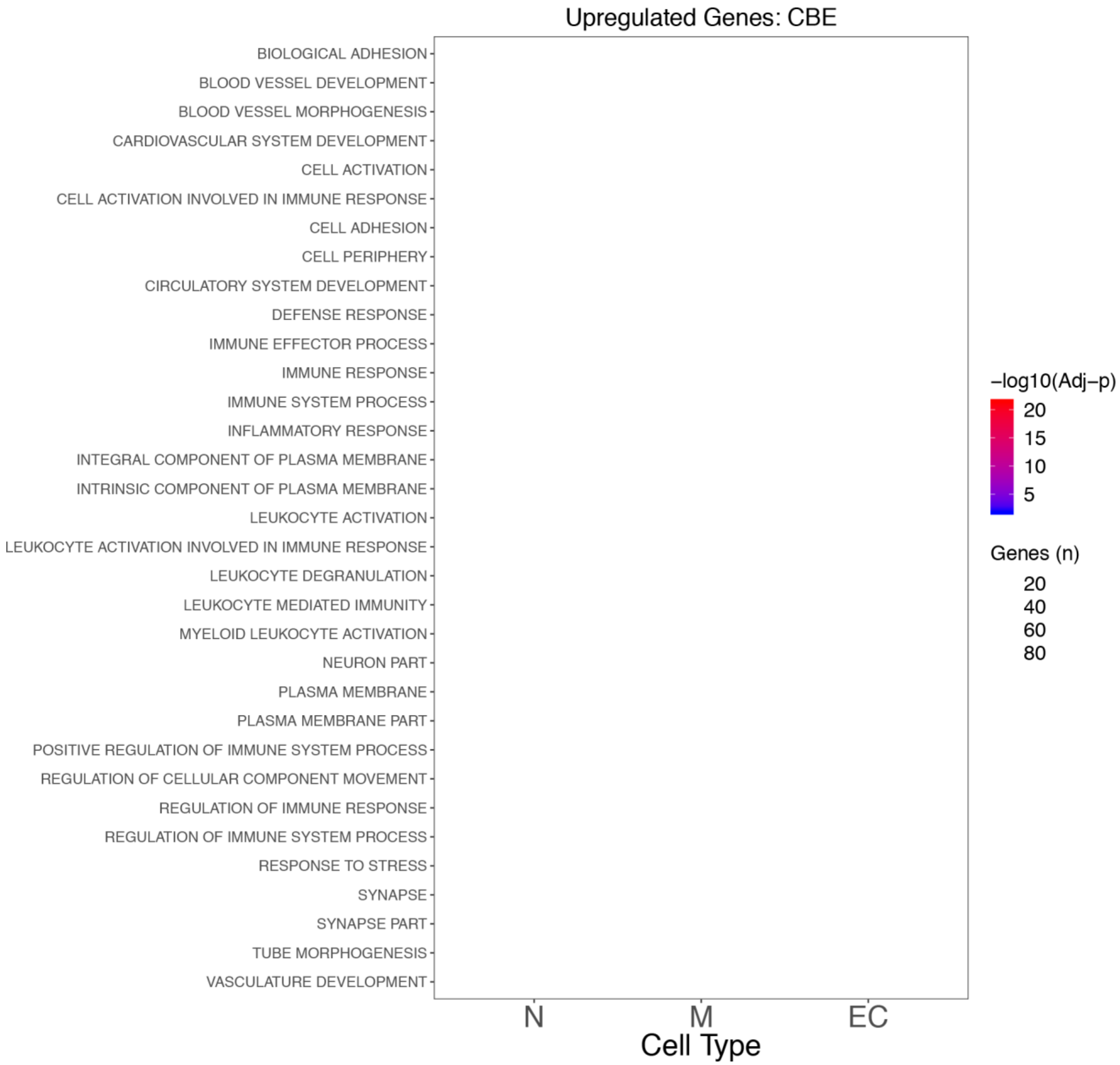

Supplementary Figure 7 (13 pages). Top 15 significant GO classes identified in the different cell types in each of the seven brain regions analyzed. The color scale indicates the significance (blue to red as the significance increases), whereas the size indicates the number of genes in that specific enriched class.

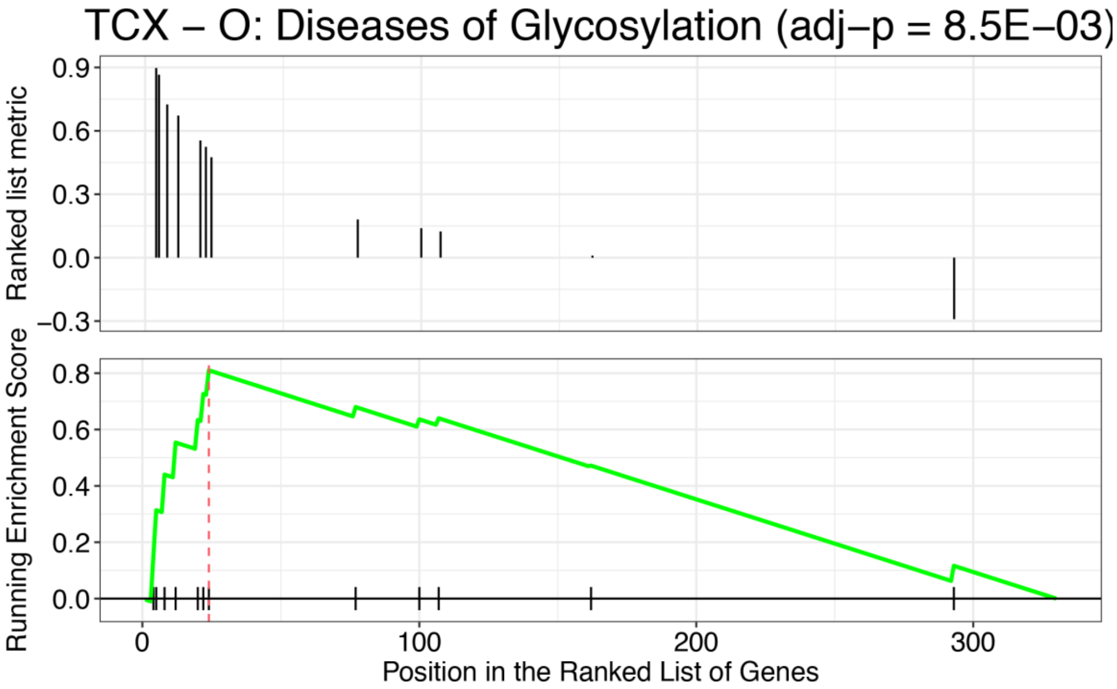
TCX – O: Diseases of Glycosylation (adj–p = 8.5E–03)
Ranked list metric
Running Enrichment Score
Position in the Ranked List of Genes

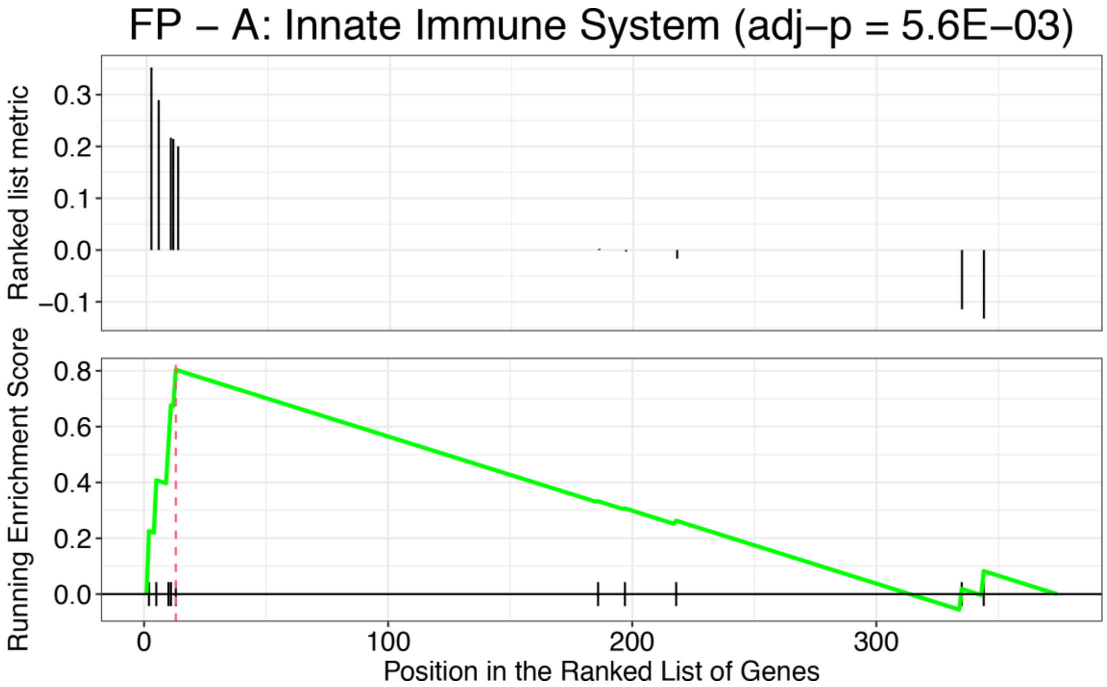
FP – A: Innate Immune System (adj–p = 5.6E–03)
Ranked list metric
Running Enrichment Score
Position in the Ranked List of Genes

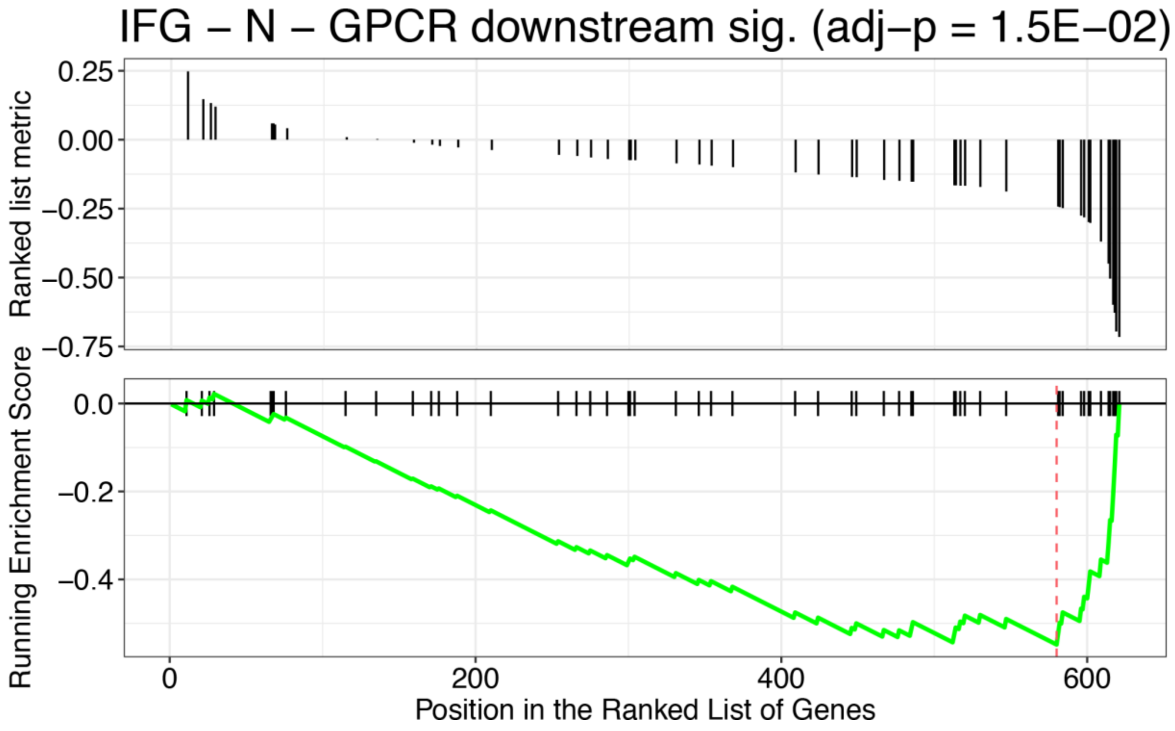
IFG – N – GPCR downstream sig. (adj−p = 1.5E−02)
Ranked list metric
0.25
0.00
−0.25
−0.50
−0.75
Running Enrichment Score
0.0
−0.2
−0.4
0
200
400
600
Position in the Ranked List of Genes

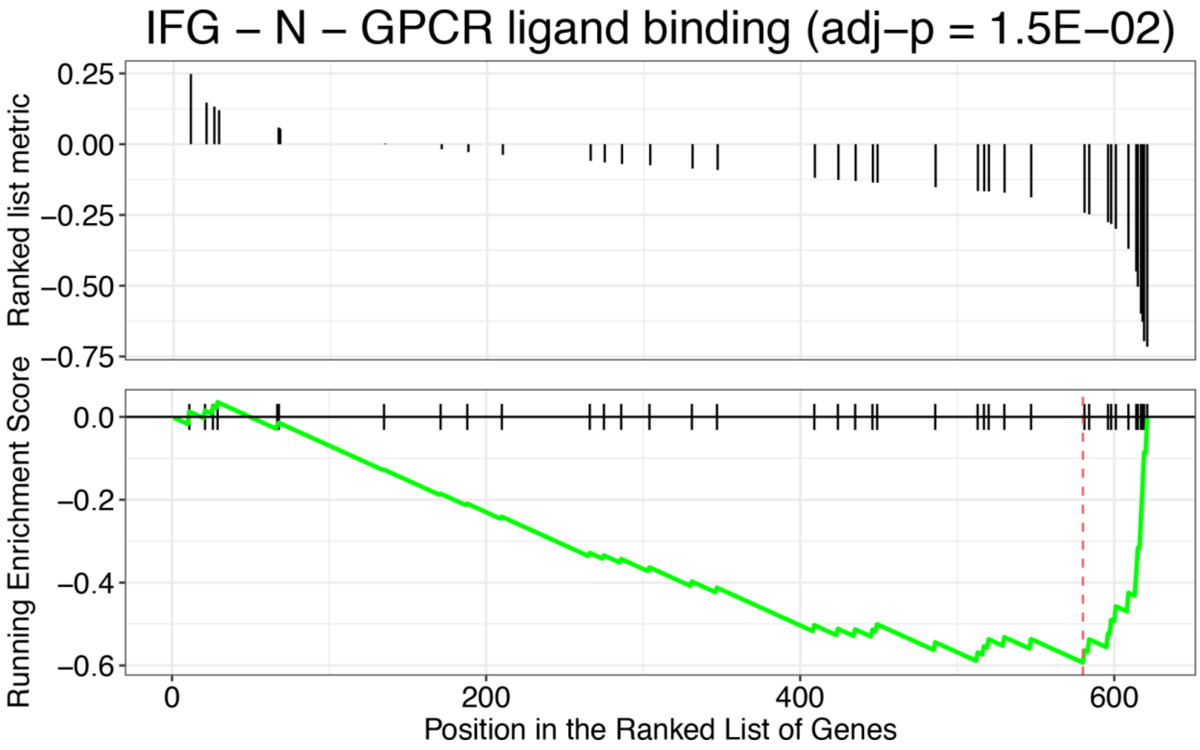
IFG – N – GPCR ligand binding (adj−p = 1.5E−02)
Ranked list metric
0.25
0.00
−0.25
−0.50
−0.75
Running Enrichment Score
0.0
−0.2
−0.4
−0.6
0
200
400
600
Position in the Ranked List of Genes

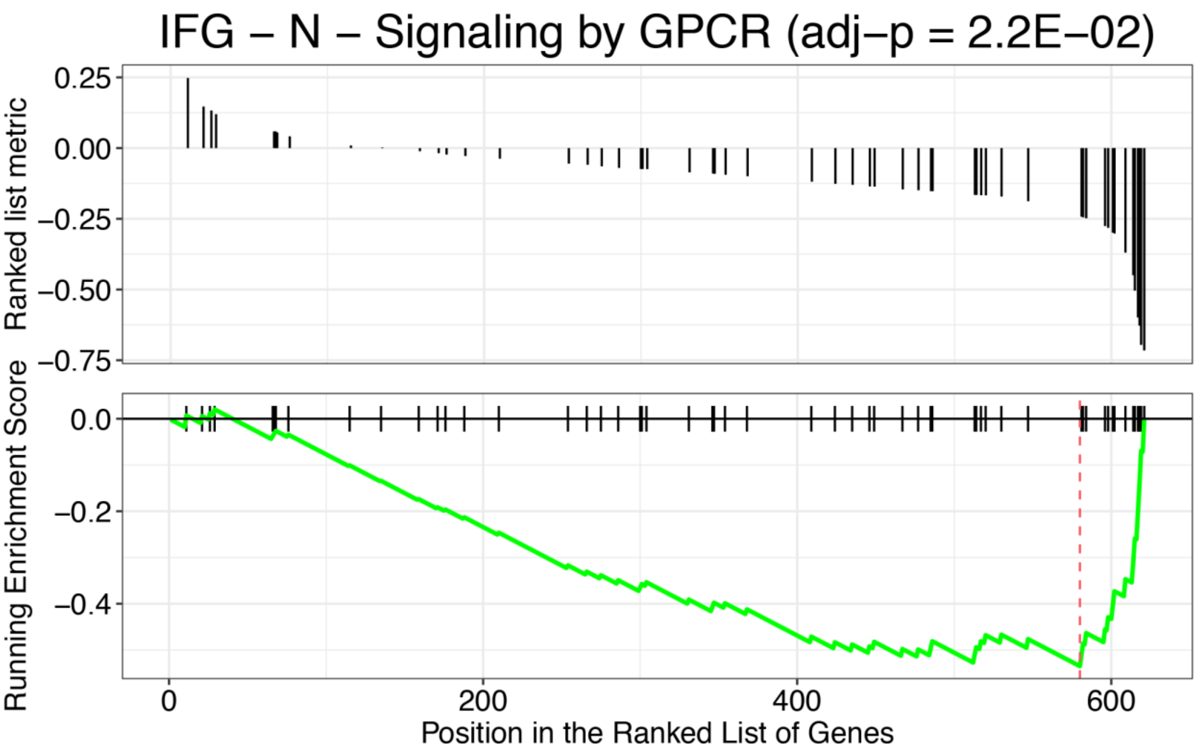

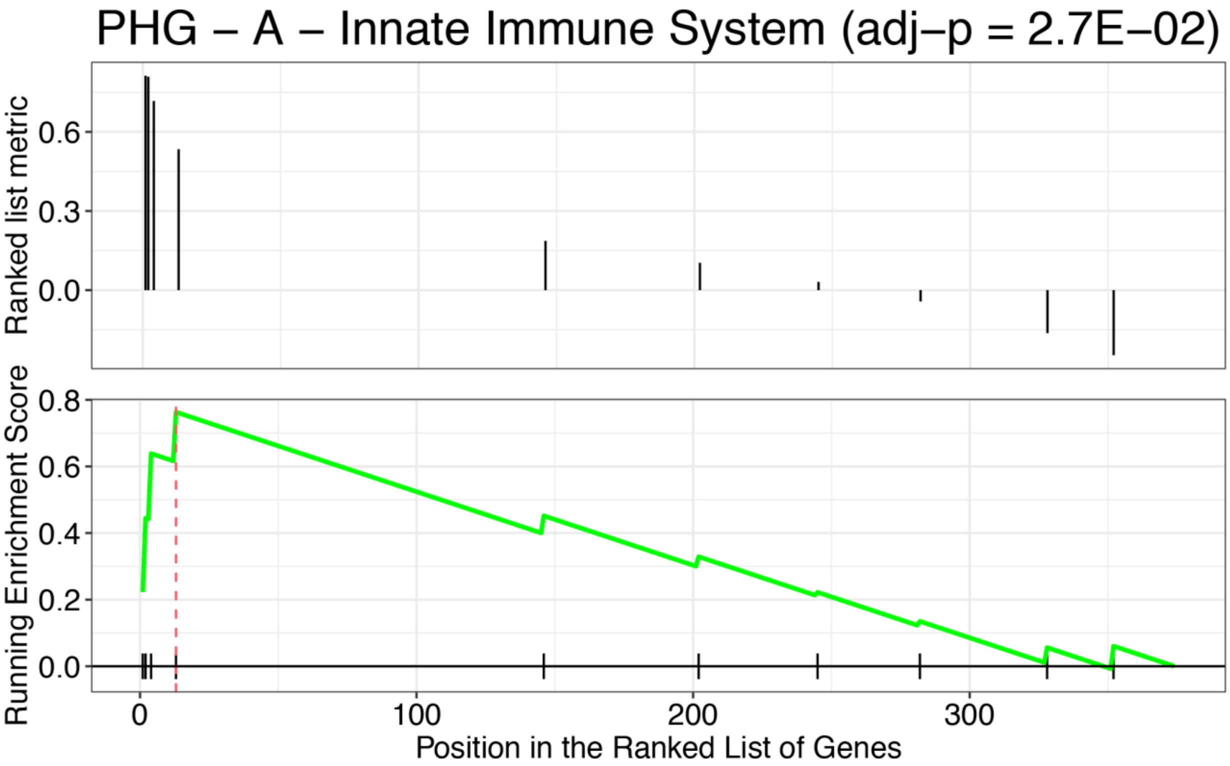

Supplementary Figure 8 (6 pages). Most relevant results from the GSEA by brain region.
